# Quality of life among French breast cancer survivors in comparison with cancer-free women: the Seintinelles study

**DOI:** 10.1186/s12905-023-02827-w

**Published:** 2024-01-03

**Authors:** Alexandra-Cristina Paunescu, Marie Préau, Cyrille Delpierre, Guillemette Jacob, Myriam Pannard, Lidia Delrieu, Marina Kvaskoff

**Affiliations:** 1 Université Paris-Saclay, UVSQ, Inserm, Gustave Roussy, CESP, 94805 Villejuif, France; 2 Pôle de Psychologie Sociale (PôPS), Unité INSERM U1296 Radiations : Défense, Santé, Environnement, Université Lyon 2, Bron, France; 3 Université de Toulouse III, CERPOP UMR1295 Inserm, UPS, Toulouse, France; 4 Association « Seintinelles. Contre Le Cancer, Tous Volontaires », Paris, France; 5 Institute for Research in bioMedicine and Epidemiology of Sport (IRMES), National Institute of Sports Expertise and Performance (INSEP), Paris, France

**Keywords:** Seintinelles, Breast cancer survivors, Cancer-free women, Health-related quality of life, Locus of control, Coping, Health literacy

## Abstract

**Background:**

Health-Related Quality of life (HRQoL) in cancer survivors can be significantly affected in the long-term by various consequences resulting from differing levels of severity of cancer and its treatments. Our objective was to identify factors associated with HRQoL in breast cancer survivors (BCSs) and cancer-free women (CFWs).

**Methods:**

We conducted a cross-sectional study in Seintinelles volunteers who answered online questionnaires between September 15, 2020 and February 5, 2021. HRQoL was measured using the World Health Organization Quality of Life–BREF questionnaire. We collected data on sociodemographic and health-related factors, lifestyle habits, coping mechanisms, locus of control, and health literacy. SAS version 9.4 statistical software was used for analyses. We performed descriptive analyses of the characteristics of the participants in each group and compared these characteristics between the two groups using the Chi^2^ test or the Student *t*-test. The adjusted means of the scores of different psychometric scales were calculated and compared using the method of least squares to fit general linear models (GLM) while adjusting for various factors. Multiple linear or multiple logistic regression models were used to assess the factors associated with WHOQOL-BREF scores, separately, in the two groups of participants.

**Results:**

The study involved 722 BCSs and 1359 CFWs aged 26–75 years. BCSs had significantly lower physical health scores and were less likely to be satisfied with their health compared to CFWs (59.5 vs. 63.2, *p* < 0.0001; and 56.5% vs. 75.2%, *p* = 0.002, respectively). In both groups, some common factors were positively associated with physical health (high financial level, being professionally active, normal BMI, good health status, alcohol consumption, higher values (> 22) of internal locus of control); or inversely associated (neurological and sleep problems, over two medical consultations/year). In BCSs, treatment by mastectomy or radiation therapy/brachytherapy, a short-time since diagnosis, current cancer therapy, and presence of sequalae were inversely associated with physical health. BCSs’ health satisfaction was diminished with lower values of coping by positive thinking (≤ 14) and seeking social support (≤ 18).

**Conclusions:**

HRQoL can be improved by developing strategies that increase internal locus of control and coping (positive thinking, problem-solving and seeking social support), and through health literacy.

**Supplementary Information:**

The online version contains supplementary material available at 10.1186/s12905-023-02827-w.

## Introduction

Breast cancer (BC) is the most common cancer among women worldwide, and the second cause of death in France after cardiovascular diseases. The number of new BC cases in 2020 in France was estimated at 58,083 (28% of cancers in women), and the estimated number of deaths from BC was of 14,183 (17.6% of cancer deaths). The same year, the prevalent number of BC cases was estimated at 236,658 (33.9% of cancers) [1].

BC is a cancer with a good prognosis. The standardized net survival in France is estimated at 97% at 1 year and 88% at 5 years, with net survivals at 5 years higher for women aged 50 and 60 (94%) and slightly lower for women aged 70 years old (92%) and 80 years old (82%) [2]. The favorable changes in survival may be explained by earlier diagnosis (especially screening), better targeted therapies, innovative treatments, and increased patient monitoring [2].

Also, BC survivors now live longer and continue to face a wide variety of long-term consequences of differing levels of severity of cancer and its treatments [3]; their quality of life (QoL) can be significantly affected in the long-term after cancer diagnosis [4–7] and the consequences of the disease can also affect their loved ones [7].

Health-related quality of life (HRQoL) is a multi-dimensional concept that covers cancer patients’ subjective perceptions of the positive and negative aspects of their symptoms, including physical, emotional, social, and cognitive functions, disease symptoms and side effects of treatment [8].

Breast cancer survivors (BCSs) with altered HRQoL often report fatigue, pain, psychological disorders such as depression, insomnia, cognitive dysfunction and negative body image [4, 9, 10]. Other factors such as beliefs about personal control are strongly associated with HRQoL in BCSs, as well as in cancer-free women (CFWs) [11]. Some coping strategies could play an important role in the persistence of fatigue [10], and health literacy has been identified as a predictor of mental HRQoL [12].

Some studies have investigated HRQoL in patients with BC at different times before and/or after cancer diagnosis or completion of treatments, using several psychometric scales [7, 13, 14], as for example, the World Health Organization Quality of Life–BREF questionnaire (WHOQOL-BREF) [15–19], but few studies have investigated the determinants of WHOQOL-BREF domains. The WHOQOL-BREF as a QoL scale makes it possible to integrate other dimensions of quality of life (QoL), apart from health (“social relationships” and “environment”), which can be applied on a longer time scale, after diagnosis and therapy.

Given the complexity of the concept of HRQoL after cancer and the unique experience of each patient going through this complex episode, along with their coping mechanisms in a more or less favorable family and social context, we proposed a cross-sectional study to estimate HRQoL among BCSs who are 1 to 10 years post-diagnosis, compared to cancer-free women in the Seintinelles study. The objectives of our study were: i) to assess and compare HRQoL between the two groups of participants (BCSs and CFWs); and ii) to identify factors associated with WHOQOL-BREF domains in each group separately, among several factors including sociodemographic and health-related factors, current lifestyle habits, and scores of personal locus of control, coping, and health literacy.

## Material and methods

### Study design

This is a cross-sectional study based on self-reported sociodemographic, health and HRQoL data among female volunteers of the French Seintinelles community. The Seintinelles is a collaborative research platform (www.seintinelles.com) with the aim of intensifying community-based cancer research, which since 2013 has included 38,240 volunteer citizens, both with and without a personal history of cancer [20], and who participate in descriptive studies (answering online questionnaires) or in interventional research related to cancer.

### Study population

The project was conducted among BCSs and CFWs Seintinelles women between September 15, 2020 and February 5, 2021. All Seintinelles participants were informed about the study via email and a message on the website and were encouraged to participate. The inclusion criteria were to be an adult, French-speaking Seintinelles volunteer woman, who provided consent to participate, and a) with a BC as first cancer that was diagnosed 1 to 10 years prior to the study, without declared recurrences, metastases or new cancer occurrence in this time interval (BCSs); or b) had no history of cancer. There was no limit to the number of volunteers who could participate in this study.

### Data collection and study measures

Participants in this study were required to answer online self-administered questions and complete several psychometric scales. To ensure answers to all questions, the possibility of answering the next question was conditioned by the answer to the previous one, with an automatic reminder when necessary. The questions covered various themes including socio-economic and familial factors (age, living status, having dependents, financial level, education level, employment status and habitat environment); current habits (tobacco smoking, alcohol consumption, physical activity); fatalistic views on cancer prevention; current health-related characteristics (Body Mass Index – BMI, health status, comorbidities, neurological problems, sleep disorders); and cancer-related characteristics in BCSs (time since diagnosis, cancer treatment types such as surgery, medical treatment or radiation therapy/brachytherapy, current therapy against cancer, presence of sequelae resulting from cancer and its treatments). All participants completed four psychometric scales (further details can be found in the Additional file 1):

1. The French-validated version [21] of the *World Organization Quality of Life Questionnaire (WHOQOL) – BREF* [22] was used to assess HRQoL of Seintinelles participants. The questionnaire includes 26 items, 24 of which are grouped into 4 domains: “physical health” (PHYSH; 7 questions), “psychological health” (PSYCH; 6 questions), “social relationships” (SOCREL; 3 questions) and “environment” (ENVIR; 8 questions), and two global items assessing the global QoL and the individual’s overall health satisfaction. The reliability of the WHOQOL-BREF is acceptable, with Cronbach's alpha coefficient above 0.65. Concurrent validity was also demonstrated globally (*p* < 0.0001), using scores for general QoL assessment, satisfaction with health, and the importance of the impact of disability on daily life [21].
2. The French-validated version [23] of the *Multidimensional Health Locus of Control Scale (MHLCS) “form A”*, an 18-item instrument [24] that measures three dimensions of the perception of control over health: “internal”, “powerful others” and “chance” (with six items each). The MHLC scales are considered reliable (Cronbach alphas ranging from 0.60 to 0.75 and test–retest stability coefficients ranging from 0.60 to 0.70) [25].
3. The French-validated version [26] of the *Brief Coping Orientation to Problems Experienced Inventory (Brief-COPE)* [27] was used to assess how people cope with a stressful event/stressful situations in their daily life [28]. The questionnaire includes 28 items grouped two by two into 14 coping strategies that then allow to define 4 factors: “positive thinking” (3 strategies), “problem solving” (2 strategies), “seeking social support” (4 strategies) and “avoidance” (5 strategies) [29]. The French version of Brief-COPE (28 items grouped into 14 coping strategies) has been validated in both a dispositional (trait coping) and a situational (state coping) format, and the results show good psychometric properties regardless of the format. In our study we used the dispositional version where the verb is in the present tense conjugation. Expected theoretical and observed structure fit adequately (in dispositional format: Chi^2^ = 606, *p* < 0.05, Root Mean Square Error of Approximation—RMSEA = 0.04; Goodness of Fit Index—GFI > 0.95; Adjusted Goodness of Fit Index—AGFI > 0.92; Root Mean Square Residual—RMR < 0.03 [26]. In addition, the 4-factor structure of the Brief-COPE demonstrated satisfactory psychometric properties; the structure showed acceptable internal consistency with all Cronbach's alpha greater than 0.6 [29].
4. The French-validated version [30] of the *European Health Literacy Survey Questionnaire* is the *short form with 16 items (HLS-EU-Q16)* of the HLS-EU-Q47 [31]. The questionnaire addresses self-reported difficulties in accessing, understanding, appraising and applying information to tasks related with making decisions in health care, disease prevention, and health promotion. The French version of the HLS-EU-Q16 has acceptable psychometric properties that allow its use in health literacy surveys, provided that the population surveyed has sufficient reading skills (preferably not below high school level) (internal consistency: Cronbach alpha coefficient of 0.81; overall Chi^2^ test p-value for Rasch model fit of 0.08) [30].

### Data processing

Age and time since diagnosis were described in tertiles. Body size was assessed through a calculated BMI (kg/m^2^). Participants were initially classified as underweight (BMI < 18.5), normal weight (BMI 18.5–24.9), overweight (BMI 25–29.9), or obese (BMI ≥ 30); the first two categories were then combined because of the very low representation of underweight women, and they were grouped under a single “normal” weight category. The scores of the various psychometric scales were calculated based on the algorithms proposed by their authors (Additional file 1) and used as either continuous or categorical variables (according to the algorithms or dichotomized according to the median).

For the WHOQOL-BREF with these four subscales (physical health, psychological health, social relationships and environment), reliability was assessed by calculating the standardized Cronbach alpha coefficients in the two groups of participants to ensure acceptable reliability with values greater than 0.70 [32] (standardized alpha Cronbach coefficients of 0.81 for BCSs and 0.79 for CFWs).

### Statistical analysis

The study first described sociodemographic characteristics, current lifestyle habits, and health-related characteristics of the participants (including cancer-related characteristics in BCSs). Next, we compared these characteristics between the two groups of participants (BCSs and CFWs) using the Chi^2^ test or the Student *t*-test according to the type of variable.

The adjusted means of the scores of different psychometric scales were calculated and compared using the method of least squares to fit general linear models (GLM) while adjusting for various factors included living status (0: alone; 1: not alone), having dependents (1: yes; 0: no), financial level (1: high; 0: low), education level (0: high school; 1: undergraduate to post-graduate degree), being professionally active (1: yes; 0: no), habitat environment (0: rural; 1: urban), age (1: 26–39; 2: 40–52; 3: 53–75 years), BMI (1: normal; 0: overweight or obese), current health status (0: good enough or lower; 1: good or very good), neurological problems (1: yes; 0: no), presence of comorbidities (cardiovascular, neurovascular diseases or diabetes; 1: yes; 0: no), consultations with a general practitioner in the last 12 months (0: ≤ 2; 1: > 2), sleep problems (1: yes; 0: no), currently smoking (1: yes; 0: no), current alcohol consumption (1: yes; 0: no), increase in physical activity level in the last 10 years (1: yes, 0: no), and fatalistic perception of cancer (1: yes; 0: no). We also calculated and compared the adjusted proportions of the categories of scores between the two groups of participants using the multiple logistic regression (multinomial logistic regression if the dependent variable had more than 2 categories).

Depending on the nature of the dependent variable (continuous or categorical), multiple linear or multiple logistic regression models were used to assess the factors associated with WHOQOL-BREF scores, separately, in the two groups of participants. The variables introduced into the multivariable models were those identified as statistically significant at the 5% level in the univariable analyses, plus other variables (*p* > 0.5) if known in relation to the outcome. The fully-adjusted model included the variables cited above, and variables from the scores of the psychometric scales dichotomized according to the median [Brief-COPE: positive thinking (1: > 14 vs. 0: ≤ 14), problem solving (1: > 11 vs. 0: ≤ 11), seeking social support (1: > 18 vs. 0: ≤ 18), and avoidance (1: > 18 vs. 0: ≤ 18); MHLCS: internal (1: > 22 vs. ≤ 22), powerful others (1: > 19 vs. 0: ≤ 19) and chance (1: > 18 vs. 0: ≤ 18); health literacy (1: sufficient > 12 vs. 0: limited ≤ 12)]. In addition, the analysis among BCSs also included the following adjusting factors: current sequelae due to cancer or its treatments (1: yes; 0: non), current cancer therapy (1: yes; 0: non), time since cancer diagnosis (1: 1–3 years; 2: 4–6 years; 3: 7–10 years), and treatments against cancer: mastectomy (1: yes; 0: non), radiation therapy (1: yes; 0: non), and medical treatment (1: yes; 0: non).

All statistical tests were two-sided, and statistical significance was defined as *p* < 0.05. All analyses were performed using the SAS statistical software package (version 9.4; SAS Institute, Cary, NC).

## Results

### Characteristics of BCSs and CFWs

A number of 2081 Seintinelles (722 BCSs and 1359 CFWs) aged between 26 and 75 years participated in this study and answered all the questions. As presented in Table 1, compared to CFWs, BCSs were significantly older (mean of 52.4 vs. 42.8 years), had fewer dependents (47.4% vs. 52.2%), and a lower level of education (high school: 21.5% vs. 11.7%), were less likely to be professionally active (67.7% vs. 80.1%), more likely to live in rural area (33.1% vs. 29.5%), and were less likely to drink alcohol (72.7% vs. 76.7%). On the other hand, compared to CFWs, BCSs were significantly less likely to report a “good or very good” health status (57.2% vs. 80.9%), and were two time more numerous to declare comorbidities (cardiovascular, neurovascular disease or diabetes; 21.9% vs. 11%). Compared to CFWs, BCSs were also significantly less likely to have “good or very good” QoL (78% vs. 83.5%), were less likely to be “satisfied or very satisfied” with his life (56.5% vs. 75.2%), but on the other hand, BCSs were more like to report a “sufficient” health literacy compared to CFWs (54% vs. 44.9%) (Table 2). Regarding BCSs, only a third were still under BC treatment (33.4%), and the majority reported experiencing sequelae due to cancer or its treatments (73.6%) (Table 1).

**Table 1:** Characteristics and lifestyle habits of breast cancer survivors and cancer-free women; the Seintinelles Study

**Characteristics**		**Breast cancer** **survivors (*n* = 722)**	**Cancer-free** **women (*n* = 1359)**	***p*-Value**
**Sociodemographic characteristics**				
		**Mean (SD)**	**Mean (SD)**	
		**Min – Max [Median]**	**Min – Max [Median]**	**Student *t*-test**
**Age (years)**		52.4 (10.1)	42.8 (11.9)	< .0001
		26–75 [53]	26–75 [41]	
		***N* (%)**	***N* (%)**	**Chi**^**2**^
	26 – 39 years	85 (11.77)	643 (47.31)	< .0001
	40 – 52 years	259 (35.87)	391 (28.77)	
	53 – 75 years	378 (52.35)	325 (23.91)	
**Living alone**	Yes	170 (23.55)	283 (20.82)	0.1521
	No^a^	552 (76.45)	1076 (79.18)	
**Dependents**^**b**^	Yes	342 (47.37)	710 (52.24)	0.0342
	No	380 (52.63)	649 (47.76)	
**Financial level**	Comfortable or very comfortable	358 (49.59)	717 (52.76)	0.1677
	Neither comfortable nor in difficulty or lower^c^	364 (50.42)	642 (47.24)	
**Education level (years of study)**	High school (≤ 12)	155 (21.47)	159 (11.70)	< .0001
	Undergraduate to post-graduate degree (13–17)^d^	567 (78.53)	1200 (88.30)	
**Currently employed**	Yes	489 (67.73)	1088 (80.06)	< .0001
	No	233 (32.27)	271 (19.94)	
**Habitat environment**	Rural	239 (33.10)	401 (29.51)	0.0907
	Urban	483 (66.90)	958 (70.49)	
**Anthropometry**				
**BMI (kg/m**^**2**^**)**	Normal (< 25)	492 (68.14)	947 (69.68)	0.4692
	Overweight or obese (≥ 25)	230 (31.86)	412 (30.32)	
**Lifestyle habits**				
**Current smoker**	Yes	62 (8.59)	130 (9.57)	0.4628
	No	660 (91.41)	1229 (90.43)	
**Current alcohol consumption**	Yes	525 (72.71)	1042 (76.67)	0.0462
	No	197 (27.29)	317 (23.33)	
**Increase in physical activity level in the last 10 years**	Yes	354 (49.03)	472 (34.73)	< .0001
	No	368 (50.97)	887 (65.27)	
**Fatalistic perception of cancer**^**e**^	Yes	243 (33.66)	306 (22.52)	< .0001
	No	479 (66.34)	1053 (77.48)	
**Health-related characteristics**				
**Current health status**	Good or very good	413 (57.20)	1099 (80.87)	< .0001
	Good enough or lower^f^	309 (42.80)	260 (19.13)	
**Number of consultations with a general practitioner in the last 12 months**^**g**^	≤ 2	422 (58.45)	810 (59.60)	0.6102
	> 2	300 (41.55)	549 (40.40)	
**Current sleep problems**^**h**^	Yes	186 (25.76)	310 (22.81)	0.1326
	No	536 (74.24)	1049 (77.19)	
**Diseases diagnosed in the last 10 years**				
**Presence of comorbidities**^**i**^	Yes	158 (21.88)	149 (10.96)	< .0001
	No	564 (78.12)	1210 (89.04)	
**Neurological problems**^**j**^	Yes	220 (30.47)	367 (27.01)	0.0945
	No	502 (69.53)	992 (72.99)	
**Cancer-related characteristics**				
**Treatments received**				
**Mastectomy**	Yes	269 (37.26)		
	No	453 (62.74)		
**Radiation therapy**^**k**^	Yes	655 (90.72)		
	No	67 (9.28)		
**Drugs**^**l**^	Yes	514 (71.19)		
	No	208 (28.81)		
**Current cancer therapy**	Yes	241 (33.38)		
	No	481 (66.62)		
**Time since diagnosis (years)**	1–3 years	259 (35.87)		
	4–6 years	255 (35.32)		
	7–10 years	208 (28.81)		
**Persistent sequelae due to cancer and its treatments**	Yes	531 (73.55)		
	No	191 (26.45)		

^a^Living with partner or with a family member

^b^Children or other dependents

^c^Includes: “in difficulty or in great difficulty” BCSs: *n* = 29 (4.02%) and CFWs: *n* = 57 (4.19%) and “neither comfortable nor in difficulty” BCSs: *n* = 335 (46.40%) and CFWs: *n* = 585 (43.05%)

^d^Education level: “undergraduate to graduate degree” (13–17 study years): BCSs: *n* = 471 (65.24%), CFWs: *n* = 964 (70.93%); and “post-graduate degree” (> 17 study years): BCSs: *n* = 96 (13.30%), CFWs: *n* = 236 (17.37%)

^e^Corresponds to wrong answers to the question: “Cancer cannot be avoided”

^f^Includes: “Bad or very bad”: BCSs: *n* = 39 (5.40%) and CFWs: *n* = 43 (3.16%); and “Good enough”: BCSs: *n* = 270 (37.40%) and CFWs: *n* = 217 (15.97%)

^g^Among BCSs, apart from any medical visit related to cancer

^h^Sleep problems: sleep < 7 h at night in the week, with difficulty falling asleep or sleep interruptions during the night

^i^Comorbidities: cardiovascular disease, neurovascular disease or diabetes

^j^Neurological disorders: Parkinson disease, depression, psychological disorders requiring treatment, migraine, memory problems requiring treatment, other neurological disease

^k^Radiation therapy or brachytherapy

^l^Medical cancer treatments: chemotherapy, immunotherapy, hormonotherapy, targeted therapy or other drug therapy

**Table 2:** Comparisons of adjusted means of psychometric scale scores between breast cancer survivors and cancer-free women

	**Breast cancer survivors (*n* = 722)**	**Cancer-free women (*n* = 1359)**	***p*-Value**
	**Adjusted mean**^**a**^** [CI 95%]**	**Adjusted mean**^**a**^** [CI 95%]**	
**WHOQOL-BREF**			
**WHOQOL: physical health [PHYSH]**	59.45 [57.95; 60.96]	63.24 [61.68; 64.80]	< .0001
**WHOQOL: psychological health [PSYCH]**	57.96 [56.28; 59.63]	58.14 [56.40; 59.88]	0.8026
**WHOQOL: social relationships [SOCREL]**	54.59 [52.53; 56.66]	55.12 [52.98; 57.27]	0.5552
**WHOQOL: environment [ENVIR]**	67.55 [66.22; 68.88]	67.20 [65.82; 68.58]	0.5445
**MHLCS – Form A**			
**MHLCS: internal**	21.58 [21.19; 21.97]	22.20 [21.79; 22.61]	0.0003
**MHLCS: powerful others**	19.73 [19.24; 20.23]	18.90 [18.38; 19.42]	0.0001
**MHLCS: chance**	18.55 [18.04; 19.05]	18.28 [17.76; 18.81]	0.2261
**Brief-COPE**			
**BCOPE: positive thinking**	14.78 [14.41; 15.16]	13.58 [13.19; 13.96]	< .0001
**BCOPE: problem solving**	10.76 [10.46; 11.07]	10.41 [10.09; 10.73]	0.0087
**BCOPE: seeking social support**	17.05 [16.56; 17.53]	17.24 [16.74; 17.74]	0.3651
**BCOPE: avoidance**	17.71 [17.34; 18.09]	18.59 [18.20; 18.98]	< .0001
**HLS-EU-Q16**^**b**^	12.16 [11.79; 12.52]	11.92 [11.54; 12.31]	0.1426

^a^Adjusted means and 95% confidence intervals; adjusted for: living status (0: alone; 1: not alone), having dependents (1: yes; 0: no), financial level (1: high; 0: lower), education level (0: high school; 1: undergraduate to post-graduate degree), being professionally active (1: yes; 0: no), habitat environment (0: rural; 1: urban), age (1: 26–39; 2: 40–52; 3: 53–75 years), BMI (1: normal; 0: overweight or obese), current health status (0: good enough or lower; 1: good or very good), neurological problems (1: yes; 0: no), presence of comorbidities (cardiovascular, neurovascular diseases or diabetes; 1: yes; 0: no), consultations with a general practitioner in the last 12 months (0: ≤ 2; 1: > 2), sleep problems (1: yes; 0: no), currently smoking (1: yes; 0: no), current alcohol consumption (1: yes; 0: no), increase in physical activity level in the last 10 years (1: yes, 0: no), and fatalistic perception of cancer (1: yes; 0: no)

^b^*N* = 1817 in whom the scores could be calculated (678 BCSs and 1139 CFWs) / 2081

### Psychometric scales scores of BCSs and CFWs

Comparisons of the unadjusted average scores between the two groups of participants are provided in Additional file 2, while the adjusted means are presented in Table 2.

Regarding WHOQOL-BREF, significant differences were only observed in the PHYSH subscale, where BCSs had a lower (“worser”) adjusted mean score compared to CFWs (59.5 vs. 63.2) (Table 2).

Regarding MHLCS, the adjusted mean scores for two subscales (“internal” and “powerful others”) showed significant differences between the two groups of participants. The “internal” dimension was lower in BCSs compared to CFWs (21.6 vs 22.2), and the perception control over health corresponding to “powerful others” was higher in BCSs compared to CFWs (19.7 vs. 18.9) (Table 2).

Regarding the Brief-COPE adjusted mean scores: “positive thinking”, “problem solving” and “avoidance”, showed significant differences between the two groups. BCSs had higher adjusted mean scores for “positive thinking” and “problem solving”, and lower scores for “avoidance” (14.7 vs. 13.6; 10.8 vs. 10.4; and 17.7 vs. 18.6, respectively) compared to CFWs (Table 2).

Comparisons of proportions of scores (unadjusted and adjusted) between BCSs and CFWs are given in Table 3 and Additional file 3.

**Table 3:** Comparisons of proportions of psychometric scale scores between breast cancer survivors and cancer-free women

Characteristics	Breast cancer survivors (*n* = 722)	Cancer-free women (*n* = 1359)	Chi^2 ^*p*-Value	Adjusted *p*-Value^a^
	***N* (%)**	***N* (%)**		
**WHOQOL-BREF: Global QoL**				
**Poor or very poor**^**b**^	28 (3.88)	58 (4.27)	0.0014	0.3307
**Neither poor nor good**	131 (18.14)	167 (12.29)		
**Good or very good**^**c**^	563 (77.98)	1134 (83.44)		
**WHOQOL-BREF: Health satisfaction**				
**Dissatisfied or very dissatisfied**^**d**^	113 (15.65)	140 (10.30)	< .0001	0.0019
**Neither satisfied nor satisfied**	201 (27.84)	197 (14.50)		
**Satisfied or very satisfied**^**e**^	408 (56.51)	1022 (75.20)		
**Health literacy (HLS-EU-Q16)**				
**Inadequate (0–8)**	73 (10.77)	158 (13.87)	< .0001	< .0001
**Problematic (9–12)**	239 (35.25)	470 (41.26)		
**Sufficient (13–16)**	366 (53.98)	511 (44.86)		
**Don’t know/ Not applicable**	220 (16.19)	44 (6.09)		

^a^Models adjusted for same variables as in Table 2, footnote a

^b^ “Very poor”: BCSs: *n* = 2 (0.28%), CFWs: *n* = 4 (0.29%); “Poor”: BCSs: *n* = 26 (3.60%), CFWs: *n* = 54 (3.97%)

^c^ “Good”: BCSs: *n* = 455 (63.02%), CFWs: *n* = 811 (59.68%); “Very good”: BCSs: *n* = 108 (14.96%), CFWs: *n* = 323 (23.77%)

^d^ “Very dissatisfied”: BCSs: *n* = 5 (0.69%), CFWs: *n* = 9 (0.66%); “Dissatisfied”: BCSs: *n* = 108 (14.96%), CFWs: *n* = 131 (9.64%)

^e^ “Satisfied”: BCSs: *n* = 355 (49.17%), CFWs: *n* = 744 (54.75%); “Very satisfied”: BCSs: *n* = 53 (7.34%), CFWs: *n* = 278 (20.46%)

Compared to CFWs, BCSs were significantly less likely to be “satisfied or very satisfied” with their health compared to CFWs (56.5% vs. 75.2%). They were also more likely to report a “sufficient” health literacy (HL scores > 12; 54% vs. 44.9%). Regarding their assessment of global QoL, even if the proportion of BCSs considering it “good or very good” was lower compared to CFWs (78% vs. 83.4%), this difference was not statistically significant in adjusted models (Table 3 and Additional file 3).

### Factors associated with scores of HRQoL in BCSs

#### WHOQOL-BREF scores (Table 4; Additional file 4)

**Table 4:** Factors associated with WHOQOL-BREF domains in breast cancer survivors and cancer-free women

**Variable**	**Breast cancer survivors (** ***n*** ** = 722)**				**Cancer-free women (** ***n*** ** = 1359)**			
	**WHOQOL-BREF**				**WHOQOL-BREF**			
	**Physical health**	**Psychological health**	**Social relationships**	**Environment**	**Physical health**	**Psychological health**	**Social relationships**	**Environment**
	**β coefficient** ^**a**^	**β coefficient** ^**a**^	**β coefficient** ^**a**^	**β coefficient** ^**a**^	**β coefficient** ^**b**^	**β coefficient** ^**b**^	**β coefficient** ^**b**^	**β coefficient** ^**b**^
**Intercept**	63.433	61.816	56.943	62.412	58.455	48.593	46.085	57.333
**Living status (not living alone)** ^**c**^	-0.306	2.129	3.727*	1.634	-0.744	2.786**	5.211***	2.299**
**Dependents (yes)**	0.037	-1.050	-0.141	-1.392	-1.409	1.324	-2.030	-1.257
**Financial level (high)**	3.964***	4.065**	1.862	7.204***	1.587*	1.944*	0.003	6.267***
**Education level (13–17 years)**	-3.107*	-1.464	-1.012	0.414	-1.472	0.861	-2.023	1.467
**Professionally active (yes)**	3.116**	0.299	0.032	0.792	4.426***	4.168***	2.554*	1.267
**Habitat environment (urban)**	0.182	-0.837	-0.099	-1.345	-0.281	0.209	1.030	-0.171
**Age (40–52 years)**	-3.011	-4.529**	-3.394	-2.010	-0.333	-1.392	-0.592	-0.452
**Age (53–75 years)**	-2.805	-3.545	-4.223	-1.044	-0.270	2.894**	0.511	1.589
**BMI (normal)**	2.875**	0.195	-1.297	-1.250	1.626*	2.344**	0.159	0.324
**Current health status (good)**	10.828***	7.602***	5.211**	3.697***	12.814***	6.537***	5.985***	5.052***
**Neurological problems** ^**d**^ ^**, e**^ ** (yes)**	-3.776**	-3.406**	-3.131*	-1.568	-5.248***	-3.220***	-1.113	-1.320
**Presence of comorbidities** ^**e**^ ^**, f**^ ** (yes)**	-2.126	-1.593	-0.169	-0.235	-3.542**	0.036	0.291	0.623
**Consultations with a general practitioner** ^**g**^ ** (> 2)**	-3.604**	-2.124*	-3.060*	0.230	-3.715***	-1.546*	-0.716	-0.438
**Currently smoking (yes)**	1.183	0.655	-1.400	-0.705	1.929	-0.060	0.415	-1.348
**Current alcohol consumption (yes)**	2.693*	0.896	1.570	2.027*	2.615**	-0.744	1.241	1.162
**Increased physical activity level (yes)**	1.358	1.561	-0.083	0.894	2.813***	1.364	0.708	1.226
**Sleep problems (yes)**	-2.258*	-1.837	-5.421**	-3.059**	-5.219***	-4.333***	-3.268**	-2.464**
**Fatalistic opinion about cancer** ^**h**^ ** (yes)**	0.921	1.898	2.263	0.576	0.642	0.426	0.592	0.890
**Brief-COPE: positive thinking** ^**i**^ ** (> 14)**	1.022	4.507***	3.689**	3.930***	2.280**	6.005***	6.322***	1.986**
**Brief-COPE: problem solving** ^**i**^ ** (> 11)**	2.933**	5.168***	2.166	2.481**	1.022	4.320***	0.142	0.921
**Brief-COPE: seeking social support** ^**i**^ ** (> 18)**	1.859	2.056*	4.412**	1.569	0.119	2.125**	4.337***	2.200**
**Brief-COPE** **: ** **avoidance** ^**i**^ ** (> 18)**	-1.242	-5.943***	-2.985*	-1.873*	-2.686**	-5.397***	-3.062**	-1.143
**MHLCS** **: ** **internal** ^**i**^ ** (> 22)**	2.399*	0.318	0.343	1.613	1.471*	0.343	0.554	1.185
**MHLCS: powerful others** ^**i**^ ** (> 19)**	-0.629	1.336	-0.129	-0.254	0.979	0.779	2.273*	0.694
**MHLCS** **: ** **chance** ^**i**^ ** (> 18)**	-1.799	-4.463***	-0.904	-0.975	-0.475	-1.218	-0.082	-0.802
**Health literacy (HLS-EU-Q16) (sufficient > 12)**	0.649	0.763	-0.084	2.160**	0.720	0.810	3.002**	1.386*
**Current sequelae of cancer (yes)**	-3.628**	-1.464	-0.656	-0.263				
**Current cancer treatment (yes)**	-4.192**	1.999	1.968	1.146				
**Response time since diagnosis (1–3 years)**	-3.494**	-3.195*	-3.483*	-2.065				
**Response time since diagnosis (4–6 years)**	-1.265	-2.446	-3.765*	-0.331				
**Mastectomy (yes)**	-3.383**	-1.848	0.950	-1.155				
**Radiation therapy** ^**j**^ ** (yes)**	-4.209*	-0.773	0.483	0.996				
**Medical treatment**^**k**^ **(yes)**	3.478**	-0.627	-0.058	-1.058				

^*^p < 0.05

^**^p < 0.01

^***^p < 0.0001

^a^β coefficient of variables in multiple linear regression model. Models adjusted for same variables as in Table 2, footnote a, and additionally for Brief-COPE: positive thinking (1: > 14 vs. 0: ≤ 14); Brief-COPE: problem solving (1: > 11 vs. 0: ≤ 11); Brief-COPE: seeking social support (1: > 18 vs. 0: ≤ 18); Brief-COPE: avoidance (1: > 18 vs. 0: ≤ 18); MHLCS: internal (1: > 22 vs. ≤ 22); MHLCS: powerful others (1: > 19 vs. 0: ≤ 19); MHLCS: chance (1: > 18 vs. 0: ≤ 18); health literacy (HLS-EU-Q16) (1: sufficient > 12 vs. 0: limited ≤ 12); current sequelae due to cancer or its treatments (1: yes vs. 0: no); current therapy against cancer (1: yes vs. 0: no); time since diagnosis and questionnaire response (1: 1–3 years vs. 0: 7–10 years); time since diagnosis and questionnaire response (1: 4–6 years vs. 0: 7–10 years); mastectomy (1: yes vs. 0: no); radiation therapy (1: yes vs. 0: no); medical cancer treatment (1: yes vs. 0: no)

^b^β coefficient of variables in multiple linear regression model. Models were adjusted for the same variables as the previous one^(a)^, except for variables related to cancer (current sequelae due to cancer or its treatments; current cancer treatment; time since diagnosis and questionnaire response; mastectomy; radiation therapy; and medical cancer treatment)

^c^ Living with partner or with a family member

^d^Neurological problems: Parkinson disease, depression, psychological disorders requiring treatment, migraine, memory problems requiring treatment, other neurological disease

^e^Diagnosis in the last 10 years

^f^Comorbidities: cardiovascular disease, neurovascular disease or diabetes

^g^In the last 12 months; apart from consultations related to cancer in BCSs

^h^Agree that: “Cancer cannot be avoided”

^i^Median value

^j^Radiation therapy or brachytherapy

^k^Medical therapy: chemotherapy, hormone therapy, immunotherapy, targeted therapy or other drug therapy

##### *Physical health*

The factors significantly positively associated with PHYSH scores were: a high financial level, being professionally active, a normal BMI and a good health status, have received medical treatment for cancer, current alcohol consumption, a Brief-COPE “problem solving” score > 11, and an “internal” MHLCS score > 22. The factors significantly inversely associated with PHYSH scores were: a high education level, presence of neurological problems, more than 2 consultations with a general practitioner in the last year and sleep problems, current therapy against cancer, have had mastectomy and treatment by radiations, have cancer-related sequelae, and short time since diagnosis (1–3 years).

##### Psychological health

The factors significantly positively associated with PSYCH scores were: a good financial level, a good health status, a Brief-COPE “positive thinking” score > 14, a “problem-solving” score > 11 and a “seeking social support” score > 18. The factors significantly inversely associated with PSYCH scores were: be between 40 and 52 years old, have neurological problems, more than 2 consultations with a general practitioner in the last year, a short time since diagnosis (1–3 years), a Brief-COPE “avoidance” score > 18, and a MHLCS “chance” score > 18.

##### Social relationships

The factors significantly positively associated with SOCREL scores were: not living alone, a good health status, a Brief-COPE “positive thinking” score > 14 and a “seeking social support” score > 18. The factors significantly inversely associated with SOCREL scores were: presence of neurological problems, more than 2 consultations with a general practitioner in the last year and sleep problems, time since diagnosis (1–6 years), and a Brief-COPE “avoidance” score > 18.

##### Environment

The factors significantly positively associated with ENVIR scores were: a good financial level, a good health status, current alcohol consumption, a Brief-COPE “positive thinking” score > 14, a “problem-solving score” > 11 and a sufficient HL score (> 12). The factors significantly inversely associated with ENVIR scores were: have sleep problems and a Brief-COPE “avoidance” score > 18.

##### Appreciation of global QoL

BCSs were more likely to rate their global QoL as good if they lived in rural area and if were alcohol consumers; and they were less likely to rate their QoL as good if they had a low financial level, a poorer health status, had had a mastectomy, or had lower values of Brief-COPE “positive thinking” (≤ 14) and “seeking social support” (≤ 18), or higher values (> 18) of “chance” MHLCS scores (Table 5).

**Table 5:** Factors associated with Good Global Quality of Life and Health Satisfaction in BCSs and CFWs BCSs and CFWs

**Variable**	**Breast cancer survivors (*n* = 722)**		**Cancer-free women (*n* = 1359)**	
	**WHOQOL-BREF**		**WHOQOL-BREF**	
	**Global Quality of life**	**Health’ satisfaction**	**Global Quality of life**	**Health’ satisfaction**
	Good (*n* = 563) vs. Low (*n* = 159)	Yes (*n* = 408) vs. No (*n* = 314)	Good (*n* = 1134) vs. Low (*n* = 225)	Yes (*n* = 1022) vs. No (*n* = 337)
	**OR [CI 95%]**^**a**^	**OR [CI 95%]**^**a**^	**OR [CI 95%]**^**b**^	**OR [CI 95%]**^**b**^
**Living alone**				
**Yes**	0.63 [0.39; 1.03]	1.49 [0.95; 2.32]	0.43 [0.30; 0.62]***	0.93 [0.63; 1.37]
**No**^**c**^	Reference	Reference	Reference	Reference
**Dependents**				
**Yes**	1.12 [0.68; 1.84]	0.89 [0.59; 1.35]	1.12 [0.78; 1.60]	1.06 [0.75; 1.50]
**No**	Reference	Reference	Reference	Reference
**Financial level**				
**Low**^**d**^	0.34 [0.22; 0.55]***	0.79 [0.55; 1.15]	0.51 [0.36; 0.72]**	0.67 [0.48; 0.93]*
**High**^**e**^	Reference	Reference	Reference	Reference
**Education level (study years)**				
**High school (≤ 12)**	1.49 [0.87; 2.55]	1.29 [0.81; 2.05]	0.78 [0.48; 1.26]	1.14 [0.70; 1.86]
**Undergraduate to post-graduate degree (> 12)**	Reference	Reference	Reference	Reference
**Professionally active**				
**Yes**	Reference	Reference	Reference	Reference
**No**	0.62 [0.39; 1.01]	1.46 [0.95; 2.23]	0.56 [0.38; 0.83]**	1.02 [0.68; 1.52]
**Habitat environment**				
**Rural**	1.92 [1.19; 3.10]**	1.00 [0.68; 1.47]	1.10 [0.77; 1.58]	1.25 [0.89; 1.76]
**Urban**	Reference	Reference	Reference	Reference
**Age (years)**				
**26 – 39**	Reference	Reference	Reference	Reference
**40 – 52**	0.54 [0.25; 1.16]	0.70 [0.38; 1.27]	0.92 [0.62; 1.37]	0.70 [0.48; 1.02]
**53 – 75**	0.52 [0.23; 1.15]	0.54 [0.29; 1.03]	1.20 [0.76; 1.88]	0.93 [0.61; 1.44]
**P**_**trend**_	0.161	0.063	0.549	0.434
**Body mass index (BMI)**				
**Normal**	Reference	Reference	Reference	Reference
**Overweight or obese**	1.44 [0.90; 2.28]	0.71 [0.48; 1.04]	1.05 [0.74; 1.48]	0.81 [0.58; 1.12]
**Current health status**				
**Good enough or lower**	0.35 [0.22; 0.56]***	0.22 [0.15; 0.32]***	0.43 [0.30; 0.62]***	0.09 [0.07; 0.13]***
**Good or very good**	Reference	Reference	Reference	Reference
**Neurological problems**^**f**^^**, g**^				
**Yes**	0.68 [0.44; 1.06]	0.83 [0.56; 1.22]	0.78 [0.55; 1.11]	0.61 [0.44; 0.85]**
**No**	Reference	Reference	Reference	Reference
**Presence of comorbidities**^**g**^^**, h**^				
**Yes**	0.75 [0.44; 1.25]	0.50 [0.32; 0.79]**	1.23 [0.73; 2.05]	0.69 [0.43; 1.10]
**No**	Reference	Reference	Reference	Reference
**Consultations with a general practitioner**^**i**^				
** ≤ 2**	Reference	Reference	Reference	Reference
** > 2**	0.94 [0.61; 1.46]	0.97 [0.67; 1.40]	0.76 [0.54; 1.07]	0.68 [0.50; 0.93]*
**Currently smoking**				
**Yes**	0.86 [0.44; 1.69]	0.96 [0.51; 1.80]	1.27 [0.74; 2.15]	1.74 [1.01; 3.00]*
**No**	Reference	Reference	Reference	Reference
**Current alcohol consumption**				
**Yes**	1.90 [1.22; 2.96]**	1.36 [0.92; 2.02]	0.83 [0.57; 1.22]	1.22 [0.85; 1.74]
**No**	Reference	Reference	Reference	Reference
**Increased physical activity level**				
**Yes**	Reference	Reference	Reference	Reference
**No**	0.82 [0.54; 1.25]	0.85 [0.59; 1.20]	0.74 [0.52; 1.05]	0.60 [0.43; 0.84]**
**Sleep problems**				
**Yes**	0.64 [0.40; 1.00]	1.16 [0.77; 1.75]	0.54 [0.38; 0.77]**	0.56 [0.40; 0.79]**
**No**	Reference	Reference	Reference	Reference
**Fatalistic opinion about cancer**^**j**^				
**Yes**	1.27 [0.80; 2.00]	0.87 [0.59; 1.27]	1.19 [0.82; 1.75]	1.09 [0.75; 1.57]
**No**	Reference	Reference	Reference	Reference
**Brief-COPE: positive thinking**^**k**^				
** ≤ 14**	0.48 [0.31; 0.75]**	0.54 [0.38; 0.77]**	0.41 [0.28; 0.61]***	0.72 [0.51; 1.01]
** > 14**	Reference	Reference	Reference	Reference
**Brief-COPE: problem solving**^**k**^				
** ≤ 11**	0.96 [0.62; 1.49]	0.93 [0.64; 1.34]	1.01 [0.71; 1.44]	1.16 [0.83; 1.62]
** > 11**	Reference	Reference	Reference	Reference
**Brief-COPE: seeking social support**^**k**^				
** ≤ 18**	0.59 [0.37; 0.92]*	0.62 [0.43; 0.90]*	0.88 [0.64; 1.21]	0.81 [0.60; 1.10]
** > 18**	Reference	Reference	Reference	Reference
**Brief-COPE: avoidance**^**k**^				
** ≤ 18**	Reference	Reference	Reference	Reference
** > 18**	0.86 [0.55; 1.34]	1.18 [0.80; 1.74]	0.65 [0.46; 0.90]**	0.79 [0.58; 1.09]
**MHLCS: internal**^**k**^				
** ≤ 22**	1.17 [0.75; 1.83]	0.75 [0.52; 1.09]	1.07 [0.77; 1.48]	0.69 [0.51; 0.95]*
** > 22**	Reference	Reference	Reference	Reference
**MHLCS: powerful others**^**k**^				
** ≤ 19**	Reference	Reference	Reference	Reference
** > 19**	0.98 [0.64; 1.50]	1.02 [0.71; 1.45]	1.38 [0.97; 1.96]	1.00 [0.72; 1.39]
**MHLCS: chance**^**k**^				
** ≤ 18**	Reference	Reference	Reference	Reference
** > 18**	0.64 [0.41; 0.99]*	0.86 [0.60; 1.25]	0.72 [0.52; 1.00]	0.95 [0.70; 1.30]
**Health literacy (HLS-EU-Q16)**				
**Limited (≤ 12)**	0.81 [0.53; 1.24]	0.87 [0.61; 1.24]	0.82 [0.59; 1.15]	0.89 [0.65; 1.22]
**Sufficient (> 12)**	Reference	Reference	Reference	Reference
**Current cancer sequelae**				
**Yes**	0.83 [0.47; 1.46]	0.78 [0.50; 1.22]		
**No**	Reference	Reference		
**Current cancer treatment**				
**Yes**	1.23 [0.73; 2.07]	0.89 [0.57; 1.39]		
**No**	Reference	Reference		
**Response time since diagnosis**				
**1 – 3 years**	0.92 [0.52; 1.62]	0.75 [0.46; 1.23]		
**4 – 6 years**	1.05 [0.60; 1.84]	1.07 [0.67; 1.70]		
**7 – 10 years**	Reference	Reference		
**P**_**trend**_	0.737	0.207		
**Mastectomy**				
**Yes**	0.55 [0.35; 0.87]*	0.81 [0.55; 1.20]		
**No**	Reference	Reference		
**Radiation therapy**^**l**^				
**Yes**	0.72 [0.33; 1.57]	0.80 [0.41; 1.56]		
**No**	Reference	Reference		
**Medical treatment**^**m**^				
**Yes**	1.11 [0.66; 1.88]	1.41 [0.90; 2.21]		
**No**	Reference	Reference		

^*^p < 0.05

^**^p < 0.01

^***^p < 0.0001

^a^Odds ratios and 95% confidence intervals. Models adjusted for same variables as in Table 4, footnote a

^b^Odds ratios and 95% confidence intervals. Models adjusted for same variables as in Table 4, footnote b

^c^Living with partner or with a family member

^d^ “Neither comfortable nor in difficulty” or lower

^e^ “Comfortable and very comfortable”

^f^Neurological problems: Parkinson disease, depression, psychological disorders requiring treatment, migraine, memory problems requiring treatment, other neurological disease

^g^Comorbidities: cardiovascular disease, neurovascular disease or diabetes

^h^Diagnosis in the last 10 years

^i^In the last 12 months; apart from consultations related to cancer in BCSs

^j^Agree that: “Cancer cannot be avoided”

^k^Median value

^l^Radiation therapy or brachytherapy

^m^Medical therapy: chemotherapy, hormone therapy, immunotherapy, targeted therapy or other drug therapy

##### Health satisfaction

BCSs were less likely to be satisfied with their health if they had a poorer health status, had comorbidities, and if they had lower values of the Brief-COPE “positive thinking” and “seeking social support” scores (Table 5).

### Factors associated with scores of HRQoL in CFWs

#### WHOQOL-BREF scores (Table 4; Additional file 5)

##### Physical health

The factors significantly positively associated with PHYSH scores were: a good financial level, being professionally active, having a normal BMI and a good general health status, current alcohol consumption, an increased physical activity level, a Brief-COPE “positive thinking” score > 14, and an “internal” MHLCS score > 22. The factors significantly inversely associated with PHYSH scores were: having neurological problems, comorbidities, more than 2 consultations with a general practitioner in the last year, sleep problems, and a Brief-COPE “avoidance” score > 18.

##### Psychological health

The factors significantly positively associated with high scores of PSYCH were: not living alone, a good financial level, being professionally active, age between 53 and 75 years, having a normal BMI and a good general health status, a Brief-COPE “positive thinking” score > 14, “problem solving” score > 11 and a “seeking social support” score > 18. The factors significantly inversely associated with PSYCH scores were: having neurological problems, more than 2 consultations with a general practitioner in the last year, sleep problems, and a Brief-COPE “avoidance” score > 18.

##### Social relationships

The factors significantly positively associated with SOCREL scores were: not living alone, being professionally active, a good health status, a Brief-COPE “positive thinking” score > 14 and a “seeking social support” > 18, a MHLCS “powerful others” score > 19, and a sufficient HL score (> 12). The factors significantly inversely associated with SOCREL scores were: sleep problems, and a Brief-COPE “avoidance” score > 18.

##### Environment

The factors significantly positively associated with ENVIR scores were: not living alone, a good financial level, a good health status, a Brief-COPE “positive thinking” score > 14, a “seeking social support” score > 18, and a sufficient HL score (> 12). Sleep problems were significantly inversely associated with ENVIR scores.

##### Appreciation of global QoL

CFWs were less likely to rate their global QoL as good if they lived alone, had a low financial level, were no longer professionally active, had a poorer health status or sleep problems, or if they had lower values (≤ 14) of Brief-COPE “positive thinking” and higher values (> 18) of “avoidance” scores (Table 5).

##### Health satisfaction

CFWs were more likely to be satisfied with their health if they were smokers, and they were less likely to be satisfied if they had a low financial level, a poorer health status, neurological problems, sleep problems, if they had had more than 2 consultations with a general practitioner, if they did not increase their level of physical activity in the past 10 years, or had lower (< 22) “internal” MHLCS scores (Table 5).

## Discussion

This cross-sectional study conducted in a large sample of BCSs and CFWs Seintinelles participants shows that BCSs had a significantly lower adjusted-mean physical health (PHYSH) score than CFWs, and that the adjusted proportion of BCSs reporting being satisfied with their health was also significantly lower compared to CFWs. Generally, the same sociodemographic and general health factors identified as being associated with the WHOQOL-BREF domains in BCSs were also found in CFWs. In addition, among CFWs, age over 52 years was associated with higher PSYCH scores, increased level of physical activity was associated with higher PHYSH scores, while the presence of comorbidities was inversely associated with this domain.

### WHOQOL-BREF scores and associated factors

The mean scores of the four WHOQOL-BREF domains and the perception of global QoL measured in BCSs vary widely between studies [7, 15–18, 33]. These differences may be due to the fact that these studies were conducted in different countries/regions, in different living environments, among people of different cultures, with different education and financial levels, different age and time periods since cancer diagnosis or surgery; and presenting above all unadjusted mean scores on specific domains of WHOQOL-BREF. For example, in the study of Kluthcovsky and Urbanetz, the average PHYSH score was also significantly lower among Brazilian BCSs 5 years post-diagnosis, compared with the cancer-free group [15].

#### Sociodemographic factors

Regarding sociodemographic factors, in BC patients in Central rural India, being without a partner was inversely associated with the PSYCH and SOCREL scores, lower education was inversely associated with ENVIR scores, whereas higher income was associated with higher three scores (PSYCH, SOCREL and ENVIR), and age above 50 years was associated with higher ENVIR scores [17]. Also, in Serbian BCSs, higher education level was associated with higher all the four WHOQOL-BREF domains; marital status (living not alone) was associated with higher SOCREL and ENVIR scores; while age was inversely associated with PSYCH, SOCREL and ENVIR scores [33]. Our results concerning a good financial level, not living alone and age, are broadly consistent with those of these studies. Furthermore, in our study, be professionally active was associated with higher PHYSH scores.

#### Health-related factors

Regarding factors related to health in general or to BC in particular, it has been reported that the presence of comorbidities was inversely associated with scores of all the WHOQOL-BREF domains, that the number of years since BC surgery was associated with higher PHYSH, PSYCH and ENVIR scores, and that compared to a classical mastectomy, a breast-conserving or mastectomy with breast reconstruction were associated with higher PHYSH scores [33]. The negative role of mastectomy on WHOQOL-BREF has also been reported by Marinkovic et al.; in this study, Serbian patients with radical mastectomy had worse scores in all four domains compared to women with conservative surgery [19]. Some other studies have shown that patients with BC in active treatment or with metastatic disease had worse PSYCH and SOCREL scores compared with survivors during the different follow-up periods [7], and that patients who were not included in a physical activity program after cancer treatment had worse PHYSH, PSYCH and SOCREL scores [18].

Similarly, in our study, some health problems (presence of neurological problems and sleep problems, more than two consultations in a year with a general practitioner excluding cancer) were inversely associated with certain WHOQOL-BREF scores; and on the other hand, in Seintinelles BCSs, a good health status was associated with higher all the four WHOQOL-BREF domains, and a normal BMI was associated with higher PHYSH scores. Regarding factors directly related to BC, our study showed also that being still under cancer treatment, having cancer-related sequelae, having undergone a mastectomy or radiation treatment (radiation therapy or brachytherapy), were inversely associated with PHYSH score. Compared to a long period of time (7–10 years), a shorter time since diagnosis (1–3 years) was inversely associated with PHYSH, PSYCH and SOCREL scores.

#### Health locus of control (HLC)

The mean scores of the three HLC domains measured in women with cancer vary between studies [34–37], and those of BCSs Seintinelles are integrated among these values. On the other hand, few studies have investigated HLC scores both in cancer patients and in healthy people. Women with cancer (or specifically with BC) had higher scores on all external HLC subscales, and lower internal HLC compared to healthy women [34, 35] and our results are consistent with these findings. In Seintinelles, the differences between the adjusted means between the two groups of participants were significant only for “internal” and “powerful others” subscales. That implies that Seintinelles BCSs believe that their health status is controlled more by external forces (by “powerful others” and to a lesser extent by “chance”), and less by their own actions (health behaviors), compared to CFWs. Individuals with a higher internal HLC have a greater predictive potential for better adaptation to cancer, a fact which implies that they are more likely to seek information about health-threatening conditions and to adopt healthier behaviors [24].

#### Coping Orientation to problem Experienced Inventory – Brief (Brief-COPE)

After BC diagnosis, many women used different coping strategies to adjust their lives accordingly, and these strategies can have an impact on patients’ perception about their illness, helps adapting to change, and consequently, affect their level of mental health, psychological well-being and QoL [38, 39].

As reported by Nipp et al., in patients with incurable cancer, reporting emotional support and acceptance coping strategies correlated with better QoL and mood, whereas denial and self-blame negatively correlated with these outcomes [38]. Women who respond to their BC diagnosis with passive acceptance and resignation are at significant risk for poor long-term psychological adjustment [40]. Also, it has been reported that maladaptive coping and lower social support increased adjusted odds of decline in QoL [41]. Moreover, a maladaptive coping style in the diagnostic phase was associated with worse HRQoL for 2 years after diagnosis in South Korean women with BC [42].

Regarding the “positive thinking” strategy, López et al. reported that it is one of the most commonly used by cancer patients and that it can predict better health benefits for the short and long term [43]. In our study, the comparison of the adjusted means of the scores indicates that BCSs use more efficient strategies in terms of QoL protection (more “positive thinking” and “problem solving” strategies and use less the “avoidance” strategy) compared to CFWs. However, adjusted “seeking social support” mean scores were comparable between the two groups of Seintinelles. Also, in the Turkish case–control study, Inci et al. found no significant differences in “seeking social support” between cancer patients and cancer-free people [44].

#### Health literacy (HL)

The European Health Literacy Survey conducted in eight countries (without France), shows that for the overall sample, the proportion of people with limited HL (inadequate or problematic levels measured with the HLS-EU-Q86 instrument) was of 47.6%, and that the distribution of levels differed substantially across countries [31]. As reported more recently by Baccolini et al. in their systematic review and meta-analysis concerning the Health Literacy in European union Member States, the pooled prevalence of low HL varies between 27 to 48%, depending on the literacy assessment method applied [45]. Southern, Western, and Eastern EU countries had lower HL compared to northern Europe; and for example, low HL in France was of 51% compared to 44% in Denmark [45]. In our study, the proportions of limited HL were different between the two groups of participants, lower in BCSs compared to CFWs (46.0% vs. 55.1%).

A low level of HL has been associated with poor health status [31, 46], more than one long-term illness [31], more hospitalizations, greater use of emergency care [31, 46], lower rate of mammography screening, poorer ability to demonstrate taking medications appropriately, and poorer ability to interpret labels and health messages [46]. In Chinese cancer survivors, inadequate HL was an independent predictor of poor QoL (measured by EORTC QOL-C30) and was associated with a lower level of functioning and a higher level of symptomatology or problems [47]. Quite the opposite, better HL was associated with better cognitive status, fewer depressive symptoms and chronic conditions, higher life-space mobility and better physical performance in 75-year-old Finnish men and women [48]. However, it should be noted that comparisons of our findings with the literature are generally difficult, given that different measurement instruments have been used, both for HRQoL and for HL. Added to this are the particularities linked to the populations under study (country, culture, age, socio-demographic characteristics, etc.).

As observed in our study, HLC, coping strategies and HL play important roles on all components of the WHOQOL-BREF. In Seintinelles, apart from certain particularities in BCSs or in CFWs, in both groups of participants, the PHYSH domain increased significantly with higher values (> 22) of “internal” HLC; the PSYCH domain increased with higher values of “problem solving” (> 11), “positive thinking” (> 14) and “seeking social support” (> 18), and decreased with higher values (> 18) of “avoidance”; the SOCREL domain increased with higher values of “positive thinking” and “seeking social support”, and decreased with higher values of “avoidance”; while the ENVIR domain increased with values corresponding to a sufficient HL (> 12). Thus, acting on these three factors could lead to an improvement in HRQoL in both BCSs and CFWs.

As stated in the literature, coping strategies and HLC can be changed by cognitive behavioral interventions [49]. The association between HL and HRQoL is important from a clinical and public health perspective, for interventions aimed at improving the overall QoL of cancer survivors [47]. Increasing QoL is important for improving symptom relief, care, and rehabilitation of patients [50], and on the other hand, to predict survival [51].

### Strengths and limitations

Our study has several strengths, such as the large sample size, the involvement of people who have had BC and also women without a personal history of cancer, the collection of data in many fields, the fact of having identified several factors positively or inversely associated with the QoL and the fact of having covered a long-time sequence, up to ten years after the end of the treatments. However, it also had limitations. The main methodological limitation of this study is its cross-sectional design, with all variables (dependent and explanatory) measured at the same time, such that the temporal sequence of cause and effect cannot be determined. We retrospectively collected self-reported data dating back 10 years, which may induce a recall bias. Participants were all volunteers with a high socio-economic and educational level, concerned about their health, which implies a selection bias. Another limitation is the fact that our study took place in the midst of the COVID-19 pandemic and that the QoL of the participants (in both groups) could have been affected by this event.

In conclusion, our study showed that BCSs had significantly lower levels of physical health and were less likely to be satisfied with their health than CFWs. Particular attention should be given to HLC, coping and HL as these factors can significantly influence WHOQOL-BREF, particularly among cancer survivors. Increasing the level of HL through wider dissemination of messages related to health in general and cancer risk factors in particular would help improve certain domains of WHOQOL-BREF. Also, developing cognitive-behavioral interventions to be able to modify coping strategies and the HLC of BCSs, such as increasing the “internal” locus of control as well as coping by developing “positive thinking”, “problem solving” rather than problem avoidance, and the search for the necessary social support, could make it possible to increase their HRQoL. The various aspects related to HRQoL should be discussed with BCSs and investigated, with the aim of developing strategies to ensure appropriate psychosocial and supportive care, and to improve the HRQoL in these cancer survivors. Further research involving a broader social representation of the population is needed to investigate the relationship between HL and HRQoL, to identify the causes of poor HL, and to develop intervention programs to improve it.

### Supplementary Information

**Additional file 1. **Psychometric scales used in the Seintinelles study.

**Additional file 2.** Comparisons of means of psychometric scale scores between breast cancer survivors (*n* = 722) and cancer-free women (*n* = 1359); the Seintinelles study.

**Additional file 3.** Comparisons of proportions of psychometric scale scores relative to the median between breast cancer survivors and cancer-free women; the Seintinelles study.

**Additional file 4.** Factors associated with WHOQOL-BREF domains in breast cancer survivors (*n* = 722); the Seintinelles study.

**Additional file 5.** Factors associated with WHOQOL-BREF domains in cancer-free women (*n* = 1359); the Seintinelles study.

## Data Availability

The datasets generated during and/or analyzed during the current study are not publicly available because informed consent from study participants did not cover public deposition of data, but are available from the corresponding author on reasonable request.

## Abbreviations

BC: Breast cancer
BCSs: Breast cancer survivors
CFWs: Cancer-free women
ENVIR: Environment
HL: Health literacy
HLC: Health locus of control
HRQoL: Health-related quality of life
PHYSH: Physical health
PSYCH: Psychological health
QoL: Quality of life
SOCREL: Social relationships
