# Supplementary material for: Quality of life among French breast cancer survivors in comparison with cancer-free women: the Seintinelles study
Source: BMC Womens Health. 2024 Jan 3;24:17. doi: 10.1186/s12905-023-02827-w (PMC10765881; doi:10.1186/s12905-023-02827-w)
Supplement: Supplementary file 1 — Additional file 1. Psychometric scales used in the Seintinelles study. [file 12905_2023_2827_MOESM1_ESM.docx]

**Additional file 1**

**Psychometric scales used in the Seintinelles study**

1. The ***World Organization Quality of Life Questionnaire (WHOQOL) - BREF*** was used to assess HRQoL of participants. The answers to each item are coded from 1 to 5. The average for each of 4 domains is calculated, taking into account that for three questions, scores must be reversed; then the average is multiplied by 4 in order to make domain scores comparable with the scores used in the WHOQOL-100. Subsequently, scores are transformed to a 0-100 scale using the formula: *Transformed score = (Score – 4) x (100/16)*. Higher scores denote higher quality of life (WHO - WHOQOL User Manual, 1998).

2. The ***Multidimensional Health Locus of Control Scale (MHLCS) “form A”*** is an 18-item instrument (Wallston et al., 1978) that measures three dimensions of the perception of control over health: “internal” control (“control over my own health depends on me”), “chance” (“health is determined by chance or other external factors”) and “powerful others” (“my own health is the result of the influence of others, especially medical personnel”) health locus of control (with six items each). The measurement scale is of the Likert type ranging from “1” (strongly disagree) to “6” (strongly agree). For each subscale, item responses are summed. The possible ranges vary between 6 and 36. People with higher MHNCS-Internal values are more likely to make positive behaviour changes. People with higher MHNCS-Chance values are less likely to exhibit the recommended health behaviours. Higher MHNCS-Powerful others values indicates that doctors and other people play a large role in determining the health status.

3. The French-validated version (Muller and Spitz, 2003) of the ***Brief Coping Orientation to Problems Experienced Inventory (Brief-COPE)*** (Carver, 1997) collects answers from the 28 items on a scale of 1 (not at all) to 4 (always). They allow the construction of 14 coping strategies: “active coping”, “planning”, “use of instrumental support”, “positive reframing”, “acceptance”, “use of emotional support”, “denial”, “venting”, “self-blame”, “humour”, “religion”, “self-distraction”, “substance use” and “behavioural disengagement”. Each “strategy” is the sum of the responses to two items. Subsequently, Baumstarck et al. (2017) proposed a grouping of “strategies” into 4 factors: “positive thinking” (3 strategies), “problem solving” (2 strategies), “seeking social support” (4 strategies) and “avoidance” (5 strategies). Higher scores reflect a higher tendency to implement the corresponding coping strategies.

4. The ***European Health Literacy Survey Questionnaire*** is the short form with 16 items (***HLS-EU-Q16***) of the HLS-EU-Q47 (Sørensen et al., 2013) that was validated in French (Rouquette et al., 2018). The questionnaire addresses self-reported difficulties in accessing, understanding, appraising and applying information to tasks related with making decisions in health care, disease prevention, and health promotion. Each item was rated on a four-point Likert scale (very difficult, difficult, easy, and very easy) and a “don’t know/no answer” category. Following the authors’ instructions, when scoring the HLS-EU-Q16, the categories “very difficult” and “difficult” are scored as 0, and the categories “easy” and “very easy” are scored as 1. Scale values are calculated as simple summed scores only for respondents who answered at least 14 items. Scoring varies between 0 and 16, establishing three levels of HL: inadequate (0–8), problematic (9–12), and sufficient (13–16).
