# Supplementary material for: Quality of life among French breast cancer survivors in comparison with cancer-free women: the Seintinelles study
Source: BMC Womens Health. 2024 Jan 3;24:17. doi: 10.1186/s12905-023-02827-w (PMC10765881; doi:10.1186/s12905-023-02827-w)
Supplement: Supplementary file 2 — Additional file 2. Comparisons of means of psychometric scale scores between breast cancer survivors (n = 722) and cancer-free women (n = 1359); the Seintinelles study. [file 12905_2023_2827_MOESM2_ESM.docx]

**Additional file 2**

**Comparisons of means of psychometric scale scores between breast cancer survivors (*n* = 722) and cancer-free women (*n* = 1359); the Seintinelles study**

|  | Breast cancer survivors  (*n* = 722) | | Cancer-free women  (*n* = 1359) | | *p*-Value |
| --- | --- | --- | --- | --- | --- |
|  | **Mean (SD)^a^** | **Min - Max** | **Mean (SD)^a^** | **Min - Max** | **Student *t*-Test** |
|  |  |  |  |  |  |
| WHOQOL-BREF |  |  |  |  |  |
| WHOQOL: physical health | 63.91 (16.13) | 7.14 - 100.0 | 71.53 (15.22) | 7.14 - 100.0 | <.0001 |
| WHOQOL: psychological health | 62.47 (15.93) | 4.17 - 100.0 | 64.63 (15.33) | 8.33 - 100.0 | 0.0025 |
| WHOQOL: social relationship | 58.26 (17.28) | 8.33 - 100.0 | 60.74 (18.31) | 0 - 100.0 | 0.0028 |
| WHOQOL: environment | 70.77 (12.51) | 12.50 - 100.0 | 71.68 (12.40) | 6.25 - 100.0 | 0.1111 |
|  |  |  |  |  |  |
|  |  |  |  |  |  |
| MHLCS – Form A |  |  |  |  |  |
| MHLCS: internal | 21.73 (3.58) | 8.0 - 36.0 | 22.47 (3.23) | 10.0 - 34.0 | <.0001 |
| MHLCS: powerful others | 19.32 (4.31) | 6.0 - 33.0 | 18.04 (4.14) | 6.0 - 35.0 | <.0001 |
| MHLCS: chance | 17.72 (4.66) | 6.0 - 36.0 | 17.60 (4.22) | 6.0 - 33.0 | 0.5530 |
|  |  |  |  |  |  |
|  |  |  |  |  |  |
| Brief-COPE |  |  |  |  |  |
| BCOPE: positive thinking | 14.71 (3.31) | 6.0 - 24.0 | 13.68 (3.12) | 6.0 - 24.0 | <.0001 |
| BCOPE: problem solving | 10.93 (2.58) | 4.0 - 16.0 | 10.48 (2.62) | 4.0 - 16.0 | 0.0002 |
| BCOPE: seeking social support | 17.71 (3.91) | 8.0 - 29.0 | 18.56 (4.21) | 8.0 - 32.0 | <.0001 |
| BCOPE: avoidance | 17.21 (3.10) | 10.0 - 30.0 | 18.12 (3.26) | 11.0 - 33.0 | <.0001 |
|  |  |  |  |  |  |
|  |  |  |  |  |  |
| HLS-EU-Q16^b^ | 12.36 (2.86) | 1.0 - 16.0 | 11.87 (3.00) | 0 - 16.0 | <.0001 |
|  |  |  |  |  |  |
|  |  |  |  |  |  |

^a^ Standard deviation.

^b^ *N* = 1817 on which the scores could be calculated (678 breast cancer survivors and 1139 cancer-free participants) / 2081.
