# Supplementary material for: Quality of life among French breast cancer survivors in comparison with cancer-free women: the Seintinelles study
Source: BMC Womens Health. 2024 Jan 3;24:17. doi: 10.1186/s12905-023-02827-w (PMC10765881; doi:10.1186/s12905-023-02827-w)
Supplement: Supplementary file 3 — Additional file 3. Comparisons of proportions of psychometric scale scores relative to the median between breast cancer survivors and cancer-free women; the Seintinelles study. [file 12905_2023_2827_MOESM3_ESM.docx]

**Additional file 3**

**Comparisons of proportions of psychometric scale scores relative to the median between breast cancer survivors and cancer-free women; the Seintinelles study**

| Characteristics | Breast cancer survivors  (*n* = 722) | Cancer-free women  (*n* = 1359) | Chi^2^  *p*-Value | Adjusted  *p*-Value^a^ |
| --- | --- | --- | --- | --- |
|  | ***N* (%)** | ***N* (%)** |  |  |
| WHOQOL-BREF |  |  |  |  |
| WHOQOL: physical health |  |  |  |  |
| ≤ 71.4 | 508 (70.36) | 641 (47.17) | <.0001 | <.0001 |
| > 71.4 | 214 (29.64) | 718 (52.83) |  |  |
|  |  |  |  |  |
| WHOQOL: psychological health |  |  |  |  |
| ≤ 66.7 | 458 (63.43) | 752 (55.33) | 0.0004 | 0.1333 |
| > 66.7 | 254 (36.57) | 607 (44.67) |  |  |
|  |  |  |  |  |
| WHOQOL: social relationship |  |  |  |  |
| ≤ 58.3 | 273 (37.81) | 436 (32.08) | 0.0087 | 0.4952 |
| > 58.3 | 449 (62.19) | 923 (67.92) |  |  |
|  |  |  |  |  |
| WHOQOL: environment |  |  |  |  |
| ≤ 71.9 | 421 (58.31) | 757 (55.70) | 0.2533 | 0.4048 |
| > 71.9 | 301 (41.69) | 602 (44.30) |  |  |
|  |  |  |  |  |
| WHOQOL: global QoL |  |  |  |  |
| Good quality of life |  |  |  |  |
| Yes^b^ | 159 (22.02) | 225 (16.56) | 0.0022 | 0.7350 |
| No^c^ | 563 (77.98) | 1134 (83.44) |  |  |
|  |  |  |  |  |
| WHOQOL: health satisfaction |  |  |  |  |
| Satisfied with their health |  |  |  |  |
| Yes^d^ | 408 (56.51) | 1022 (75.20) | <.0001 | 0.0016 |
| No^e^ | 314 (43.49) | 337 (24.80) |  |  |
|  |  |  |  |  |
| MHLCS – Form A |  |  |  |  |
| MHLCS: internal |  |  |  |  |
| ≤ 22 | 440 (60.94) | 721 (53.05) | 0.0006 | 0.0098 |
| > 22 | 282 (39.06) | 638 (46.95) |  |  |
|  |  |  |  |  |
| MHLCS: powerful others |  |  |  |  |
| ≤ 19 | 349 (48.34) | 867 (63.80) | <.0001 | <.0001 |
| > 19 | 373 (51.66) | 492 (36.20) |  |  |
|  |  |  |  |  |
| MHLCS: chance |  |  |  |  |
| ≤ 18 | 411 (56.93) | 775 (57.03) | 0.9643 | 0.5389 |
| > 18 | 311 (43.07) | 584 (42.97) |  |  |
|  |  |  |  |  |
| Brief-COPE |  |  |  |  |
| Brief-COPE: positive thinking |  |  |  |  |
| ≤ 14 | 349 (48.34) | 835 (61.44) | <.0001 | <.0001 |
| > 14 | 373 (51.66) | 524 (38.56) |  |  |
|  |  |  |  |  |
| Brief-COPE: problem solving |  |  |  |  |
| ≤ 11 | 399 (55.26) | 818 (60.19) | 0.0299 | 0.1014 |
| > 11 | 323 (44.74) | 541 (39.81) |  |  |
|  |  |  |  |  |
| Brief-COPE: seeking social support |  |  |  |  |
| ≤ 18 | 432 (59.83) | 668 (49.15) | <.0001 | 0.0846 |
| > 18 | 290 (40.17) | 691 (50.85) |  |  |
|  |  |  |  |  |
| Brief-COPE: avoidance |  |  |  |  |
| ≤ 18 | 498 (68.98) | 807 (59.38) | <.0001 | 0.0008 |
| > 18 | 224 (31.02) | 552 (40.62) |  |  |
|  |  |  |  |  |
| Health literacy (HLS-EU-Q16) |  |  |  |  |
| Insufficient (≤ 12)^f^ | 356 (49.31) | 848 (62.40) | <.0001 | 0.0004 |
| Sufficient (> 12) | 366 (50.69) | 511 (37.60) |  |  |
|  |  |  |  |  |

^a^ Adjusted for: living status (alone; not alone), have dependents (yes; no), financial level (high; lower), education level (high school; undergraduate to post-graduate degree), professionally active (yes; no), habitat environment (rural; urban), age (26-39; 40-52; 53-75 years), BMI (normal; overweight or obese), current health status (good enough or lower; good or very good), neurological problems (yes; no), presence of comorbidities (cardiovascular, neurovascular diseases or diabetes: yes; no), consultation with a general practitioner in the last 12 months (≤ 2; > 2), sleep problems (yes; no), current smoker (yes; no), current alcohol consumption (yes; no), increase in physical activity level in the last 10 years (yes, no), and fatalistic perception of cancer (yes; no).

^b^ Includes categories: “good” and “very good”.

^c^ Includes categories: “poor” and “very poor”.

^d^ Includes categories: “satisfied” and “very satisfied”.

^e^ Includes categories: “dissatisfied” and “very dissatisfied”.

^f^ Includes categories: “inadequate”, “problematic” and “don't know / not concerned”.
