# Supplementary material for: Quality of life among French breast cancer survivors in comparison with cancer-free women: the Seintinelles study
Source: BMC Womens Health. 2024 Jan 3;24:17. doi: 10.1186/s12905-023-02827-w (PMC10765881; doi:10.1186/s12905-023-02827-w)
Supplement: Supplementary file 4 — Additional file 4. Factors associated with WHOQOL-BREF domains in breast cancer survivors (n = 722); the Seintinelles study. [file 12905_2023_2827_MOESM4_ESM.docx]

**Additional file 4**

**Factors associated with WHOQOL-BREF domains in breast cancer survivors** **(*n* = 722); the Seintinelles study**

|  | WHOQOL: physical health^a^ | | | | | WHOQOL: psychological health^a^ | | | | | WHOQOL: social relationships^a^ | | | | | WHOQOL: environment^a^ | | | | |
| --- | --- | --- | --- | --- | --- | --- | --- | --- | --- | --- | --- | --- | --- | --- | --- | --- | --- | --- | --- | --- |
| Variable | **β** | **SE** | ***p*-Value** | **CI 95% inf.** | **CI 95% sup.** | **β** | **SE** | ***p*-Value** | **CI 95% inf.** | **CI 95% sup.** | **β** | **SE** | ***p*-Value** | **CI 95% inf.** | **CI 95% sup.** | **β** | **SE** | ***p*-Value** | **CI 95% inf.** | **CI 95% sup.** |
| Intercept | 63.433 | 3.495 | <.0001 | 56.572 | 70.294 | 61.816 | 3.713 | <.0001 | 54.525 | 69.107 | 56.943 | 4.500 | <.0001 | 48.108 | 65.778 | 62.412 | 3.013 | <.0001 | 56.497 | 68.328 |
| Living status^b^ (living not alone) | -0.306 | 1.171 | 0.7939 | -2.604 | 1.993 | 2.129 | 1.244 | 0.0875 | -0.314 | 4.571 | 3.727 | 1.507 | 0.0137 | 0.767 | 6.686 | 1.634 | 1.009 | 0.1059 | -0.348 | 3.616 |
| Dependents (yes) | 0.037 | 1.116 | 0.9737 | -2.154 | 2.228 | -1.050 | 1.186 | 0.3761 | -3.378 | 1.278 | -0.141 | 1.437 | 0.9221 | -2.962 | 2.681 | -1.392 | 0.962 | 0.1484 | -3.281 | 0.497 |
| Financial level (hight) | 3.964 | 1.006 | <.0001 | 1.990 | 5.939 | 4.065 | 1.069 | 0.0002 | 1.966 | 6.163 | 1.862 | 1.295 | 0.1510 | -0.681 | 4.405 | 7.204 | 0.867 | <.0001 | 5.502 | 8.907 |
| Education level (13-17 years) | -3.107 | 1.231 | 0.0118 | -5.524 | -0.690 | -1.464 | 1.308 | 0.2635 | -4.032 | 1.105 | -1.012 | 1.585 | 0.5234 | -4.124 | 2.100 | 0.414 | 1.061 | 0.6963 | -1.669 | 2.498 |
| Professionally active (yes) | 3.116 | 1.129 | 0.0059 | 0.899 | 5.334 | 0.299 | 1.200 | 0.8033 | -2.057 | 2.655 | 0.032 | 1.454 | 0.9827 | -2.824 | 2.887 | 0.792 | 0.974 | 0.4162 | -1.120 | 2.704 |
| Habitat environment (urban) | 0.182 | 1.026 | 0.8596 | -1.834 | 2.197 | -0.837 | 1.091 | 0.4432 | -2.978 | 1.305 | -0.099 | 1.322 | 0.9404 | -2.694 | 2.496 | -1.345 | 0.885 | 0.1290 | -3.082 | 0.393 |
| Age (40-52 years) | -3.011 | 1.608 | 0.0616 | -6.169 | 0.146 | -4.529 | 1.709 | 0.0082 | -7.884 | -1.173 | -3.394 | 2.071 | 0.1017 | -7.460 | 0.672 | -2.010 | 1.387 | 0.1476 | -4.732 | 0.712 |
| Age (53-75 years) | -2.805 | 1.703 | 0.1001 | -6.149 | 0.540 | -3.545 | 1.810 | 0.0505 | -7.099 | 0.008 | -4.223 | 2.193 | 0.0546 | -8.529 | 0.083 | -1.044 | 1.468 | 0.4775 | -3.927 | 1.840 |
| BMI (normal) | 2.875 | 1.055 | 0.0066 | 0.804 | 4.945 | 0.195 | 1.121 | 0.8617 | -2.005 | 2.396 | -1.297 | 1.358 | 0.3398 | -3.964 | 1.370 | -1.250 | 0.909 | 0.1697 | -3.035 | 0.536 |
| Current health status (good) | 10.828 | 1.096 | <.0001 | 8.677 | 12.979 | 7.602 | 1.164 | <.0001 | 5.316 | 9.887 | 5.211 | 1.411 | 0.0002 | 2.441 | 7.981 | 3.697 | 0.945 | <.0001 | 1.843 | 5.552 |
| Neurological problems^c, d^ (yes) | -3.776 | 1.062 | 0.0004 | -5.861 | -1.691 | -3.406 | 1.128 | 0.0026 | -5.621 | -1.190 | -3.131 | 1.368 | 0.0223 | -5.816 | -0.447 | -1.568 | 0.916 | 0.0873 | -3.365 | 0.230 |
| Presence of comorbidities^d, e^ (yes) | -2.126 | 1.236 | 0.0859 | -4.553 | 0.301 | -1.593 | 1.314 | 0.2256 | -4.172 | 0.986 | -0.169 | 1.592 | 0.9157 | -3.294 | 2.957 | -0.235 | 1.066 | 0.8253 | -2.328 | 1.857 |
| Consultation with a general practitioner^f^ (> 2) | -3.604 | 1.008 | 0.0004 | -5.584 | -1.624 | -2.124 | 1.071 | 0.0478 | -4.228 | -0.021 | -3.060 | 1.298 | 0.0187 | -5.609 | -0.511 | 0.230 | 0.869 | 0.7910 | -1.476 | 1.937 |
| Currently smoking (yes) | 1.183 | 1.689 | 0.4838 | -2.133 | 4.499 | 0.655 | 1.794 | 0.7153 | -2.869 | 4.178 | -1.400 | 2.175 | 0.5200 | -5.669 | 2.870 | -0.705 | 1.456 | 0.6282 | -3.564 | 2.153 |
| Current alcohol consumption (yes) | 2.693 | 1.060 | 0.0113 | 0.611 | 4.774 | 0.896 | 1.126 | 0.4268 | -1.316 | 3.107 | 1.570 | 1.365 | 0.2505 | -1.110 | 4.250 | 2.027 | 0.914 | 0.0269 | 0.233 | 3.821 |
| Increased physical activity level (yes) | 1.358 | 0.945 | 0.1512 | -0.498 | 3.214 | 1.561 | 1.005 | 0.1206 | -0.411 | 3.534 | -0.083 | 1.217 | 0.9457 | -2.473 | 2.307 | 0.894 | 0.815 | 0.2729 | -0.706 | 2.494 |
| Sleep problems (yes) | -2.258 | 1.099 | 0.0402 | -4.416 | -0.101 | -1.837 | 1.168 | 0.1160 | -4.130 | 0.455 | -5.421 | 1.415 | 0.0001 | -8.199 | -2.643 | -3.059 | 0.947 | 0.0013 | -4.919 | -1.200 |
| Fatalistic opinion about cancer^g^ (yes) | 0.921 | 1.036 | 0.3743 | -1.113 | 2.955 | 1.898 | 1.101 | 0.0852 | -0.264 | 4.060 | 2.263 | 1.334 | 0.0903 | -0.356 | 4.883 | 0.576 | 0.893 | 0.5191 | -1.178 | 2.330 |
| Brief-COPE: positive thinking^h^ (> 14) | 1.022 | 0.973 | 0.2941 | -0.889 | 2.932 | 4.507 | 1.034 | <.0001 | 2.476 | 6.537 | 3.689 | 1.253 | 0.0034 | 1.228 | 6.149 | 3.930 | 0.839 | <.0001 | 2.283 | 5.578 |
| Brief-COPE: problem solving^h^ (> 11) | 2.933 | 0.982 | 0.0029 | 1.005 | 4.860 | 5.168 | 1.043 | <.0001 | 3.120 | 7.21637 | 2.166 | 1.264 | 0.0870 | -0.316 | 4.648 | 2.481 | 0.846 | 0.0035 | 0.820 | 4.143 |
| Brief-COPE: seeking social support^h^ (> 18) | 1.859 | 0.978 | 0.0579 | -0.062 | 3.780 | 2.056 | 1.040 | 0.0483 | 0.015 | 4.098 | 4.412 | 1.260 | 0.0005 | 1.939 | 6.886 | 1.569 | 0.844 | 0.0633 | -0.087 | 3.225 |
| Brief-COPE: avoidance^h^ (> 18) | -1.242 | 1.043 | 0.2339 | -3.289 | 0.805 | -5.943 | 1.108 | <.0001 | -8.118 | -3.768 | -2.985 | 1.342 | 0.0265 | -5.620 | -0.349 | -1.873 | 0.899 | 0.0376 | -3.637 | -0.108 |
| MHLCS: internal^h^ (> 22) | 2.399 | 0.998 | 0.0165 | 0.439 | 4.359 | 0.318 | 1.061 | 0.7642 | -1.764 | 2.401 | 0.343 | 1.285 | 0.7898 | -2.181 | 2.866 | 1.613 | 0.861 | 0.0614 | -0.077 | 3.302 |
| MHLCS: powerful others^h^ (> 19) | -0.629 | 0.958 | 0.5121 | -2.510 | 1.253 | 1.336 | 1.018 | 0.1902 | -0.664 | 3.335 | -0.129 | 1.234 | 0.9166 | -2.552 | 2.294 | -0.254 | 0.826 | 0.7582 | -1.877 | 1.368 |
| MHLCS: chance^h^ (> 18) | -1.799 | 0.998 | 0.0719 | -3.758 | 0.160 | -4.463 | 1.060 | <.0001 | -6.545 | -2.381 | -0.904 | 1.285 | 0.4818 | -3.427 | 1.619 | -0.975 | 0.860 | 0.2574 | -2.664 | 0.714 |
| Health literacy (sufficient > 12) | 0.649 | 0.954 | 0.4963 | -1.223 | 2.521 | 0.763 | 1.013 | 0.4519 | -1.227 | 2.752 | -0.084 | 1.228 | 0.9458 | -2.494 | 2.327 | 2.160 | 0.822 | 0.0088 | 0.546 | 3.774 |
| Current cancer sequelae (yes) | -3.628 | 1.183 | 0.0022 | -5.950 | -1.306 | -1.464 | 1.257 | 0.2444 | -3.932 | 1.003 | -0.656 | 1.523 | 0.6670 | -3.646 | 2.335 | -0.263 | 1.020 | 0.7965 | -2.265 | 1.739 |
| Current therapy against cancer (yes) | -4.192 | 1.187 | 0.0004 | -6.523 | -1.861 | 1.999 | 1.261 | 0.1136 | -0.478 | 4.475 | 1.968 | 1.529 | 0.1984 | -1.033 | 4.969 | 1.146 | 1.023 | 0.2632 | -0.863 | 3.155 |
| Time since diagnosis (1-3 years) | -3.494 | 1.308 | 0.0077 | -6.063 | -0.926 | -3.195 | 1.390 | 0.0219 | -5.924 | -0.465 | -3.483 | 1.685 | 0.0391 | -6.790 | -0.175 | -2.065 | 1.128 | 0.0676 | -4.279 | 0.150 |
| Time since diagnosis (4-6 years) | -1.265 | 1.245 | 0.3099 | -3.710 | 1.180 | -2.446 | 1.323 | 0.0650 | -5.043 | 0.152 | -3.765 | 1.603 | 0.0191 | -6.913 | -0.617 | -0.331 | 1.074 | 0.7581 | -2.439 | 1.777 |
| Mastectomy (yes) | -3.383 | 1.051 | 0.0013 | -5.446 | -1.320 | -1.848 | 1.117 | 0.0984 | -4.040 | 0.345 | 0.950 | 1.353 | 0.4829 | -1.707 | 3.606 | -1.155 | 0.906 | 0.2025 | -2.934 | 0.623 |
| Treatment by radiations^i^ (yes) | -4.209 | 1.707 | 0.0139 | -7.561 | -0.858 | -0.773 | 1.814 | 0.6702 | -4.334 | 2.789 | 0.483 | 2.198 | 0.8261 | -3.833 | 4.799 | 0.996 | 1.472 | 0.4986 | -1.893 | 3.886 |
| Drug treatment^j^ (yes) | 3.478 | 1.194 | 0.0037 | 1.134 | 5.822 | -0.627 | 1.269 | 0.6216 | -3.118 | 1.865 | -0.058 | 1.538 | 0.9701 | -3.077 | 2.961 | -1.058 | 1.029 | 0.3046 | -3.079 | 0.964 |

^a^ Multiple linear regression model. Models were adjusted for: living status (1: not alone vs. 0: alone); dependents (1: yes vs. 0: no); financial level (1: high vs. 0: low); education level (1: undergraduate to post-graduate degree, 13-17 years vs. 0: high school, ≤ 12 years); professionally active (1: yes vs. 0: no); habitat environment (1: urban vs. 0: rural); age (1: 40-52 years vs. 0: 36-39 years); age (1: 53-75 years vs. 0: 36-39 years); BMI (1: normal vs. 0: overweight or obese); current health status (1: good or very good vs. 0: good enough or lower); neurological problems (1: yes vs. 0: no); presence of comorbidities (1: yes vs. 0: no); consultations with a general practitioner (1: > 2 vs. 0 < 2); currently smoking (1: yes vs. 0: no); current alcohol consumption (1: yes vs. 0: no); increased physical activity level (1: yes vs. 0: no); sleep problems (1: yes vs. 0: no); fatalistic opinion about cancer (1: yes vs. 0: no); Brief-COPE: positive thinking (1: > 14 vs. 0: ≤ 14); Brief-COPE: problem solving (1: > 11 vs. 0: ≤ 11); Brief-COPE: seeking social support (1: > 18 vs. 0: ≤ 18); Brief-COPE: avoidance (1: > 18 vs. 0: ≤ 18); MHLCS: internal (1: > 22 vs. ≤ 22); MHLCS: powerful others (1: > 19 vs. 0: ≤ 19); MHLCS: chance (1: > 18 vs. 0: ≤ 18); health literacy (HLS-EU-Q16) (1: sufficient > 12 vs. 0: limited ≤ 12); current sequelae due to cancer or its treatments (1: yes vs. 0: no); current therapy against cancer (1: yes vs. 0: no); time since diagnosis and questionnaire response (1: 1-3 years vs. 0: 7-10 years); time since diagnosis and questionnaire response (1: 4-6 years vs. 0: 7-10 years); mastectomy (1: yes vs. 0: no); treatment by radiations (1: yes vs. 0: no); drug treatment against cancer (1: yes vs. 0: no);.

^b^ Living with partner or with a family member.

^c^ Neurological problems: Parkinson disease; memory problems requiring consultation; depression, psychological disorders requiring treatment; migraine, or other neurological disease.

^d^ Diagnosis in the last 10 years.

^e^ Comorbidities: cardiovascular disease, neurovascular disease or diabetes.

^f^ In the last 12 months.

^g^ Agree that: “Cancer cannot be avoided”.

^h^ Median value.

^i^ Radiotherapy or brachytherapy.

^j^ Drug therapy: chemotherapy, hormone therapy, immunotherapy, targeted therapy or other drug therapy.

* *p* < 0.05; ** *p* < 0.01; *** *p* < 0.0001.
