# Supplementary material for: Quality of life among French breast cancer survivors in comparison with cancer-free women: the Seintinelles study
Source: BMC Womens Health. 2024 Jan 3;24:17. doi: 10.1186/s12905-023-02827-w (PMC10765881; doi:10.1186/s12905-023-02827-w)
Supplement: Supplementary file 5 — Additional file 5. Factors associated with WHOQOL-BREF domains in cancer-free women (n = 1359); the Seintinelles study. [file 12905_2023_2827_MOESM5_ESM.docx]

**Additional file 5**

**Factors associated with WHOQOL-BREF domains in cancer-free women (*n* = 1359); the Seintinelles study**

|  | WHOQOL: physical health^a^ | | | | | WHOQOL: psychological health^a^ | | | | | WHOQOL: social relationship^a^ | | | | | WHOQOL: environment^a^ | | | | |
| --- | --- | --- | --- | --- | --- | --- | --- | --- | --- | --- | --- | --- | --- | --- | --- | --- | --- | --- | --- | --- |
| Variable | **β** | **SE** | ***p*-Value** | **CI 95% inf.** | **CI 95% sup.** | **β** | **SE** | ***p*-Value** | **CI 95% inf.** | **CI 95% sup.** | **β** | **SE** | ***p*-Value** | **CI 95% inf.** | **CI 95% sup.** | **β** | **SE** | ***p*-Value** | **CI 95% inf.** | **CI 95% sup.** |
| Intercept | 58.455 | 2.068 | <.0001 | 54.398 | 62.512 | 48.593 | 2.184 | <.0001 | 44.309 | 52.877 | 46.085 | 2.920 | <.0001 | 40.356 | 51.814 | 57.333 | 1.870 | <.0001 | 53.663 | 61.002 |
| Living status^b^ (living not alone) | -0.744 | 0.877 | 0.3963 | -2.465 | 0.977 | 2.786 | 0.926 | 0.0027 | 0.968 | 4.603 | 5.211 | 1.239 | <.0001 | 2.781 | 7.641 | 2.299 | 0.793 | 0.0038 | 0.743 | 3.855 |
| Dependents (yes) | -1.409 | 0.748 | 0.0598 | -2.876 | 0.058 | 1.324 | 0.790 | 0.0939 | -0.225 | 2.873 | -2.030 | 1.056 | 0.0548 | -4.102 | 0.042 | -1.257 | 0.676 | 0.0634 | -2.584 | 0.070 |
| Financial level (hight) | 1.587 | 0.725 | 0.0286 | 0.166 | 3.009 | 1.944 | 0.765 | 0.0112 | 0.443 | 3.445 | 0.003 | 1.023 | 0.9979 | -2.004 | 2.010 | 6.267 | 0.655 | <.0001 | 4.982 | 7.552 |
| Education level (13-17 years) | -1.472 | 1.118 | 0.1881 | -3.666 | 0.721 | 0.861 | 1.181 | 0.4663 | -1.456 | 3.177 | -2.023 | 1.579 | 0.2003 | -5.121 | 1.075 | 1.467 | 1.011 | 0.1472 | -0.517 | 3.450 |
| Professionally active (yes) | 4.426 | 0.903 | <.0001 | 2.655 | 6.196 | 4.168 | 0.953 | <.0001 | 2.298 | 6.038 | 2.554 | 1.275 | 0.0453 | 0.054 | 5.054 | 1.267 | 0.816 | 0.1209 | -0.334 | 2.868 |
| Habitat environment (urban) | -0.281 | 0.764 | 0.7134 | -1.780 | 1.219 | 0.209 | 0.807 | 0.7959 | -1.375 | 1.792 | 1.030 | 1.080 | 0.3403 | -1.088 | 3.147 | -0.171 | 0.691 | 0.8050 | -1.527 | 1.186 |
| Age (40-52 years) | -0.333 | 0.834 | 0.6898 | -1.970 | 1.304 | -1.392 | 0.881 | 0.1145 | -3.120 | 0.337 | -0.592 | 1.178 | 0.6154 | -2.903 | 1.719 | -0.452 | 0.755 | 0.5493 | -1.932 | 1.028 |
| Age (53-75 years) | -0.270 | 0.948 | 0.7761 | -2.129 | 1.590 | 2.894 | 1.001 | 0.0039 | 0.931 | 4.857 | 0.511 | 1.338 | 0.7025 | -2.114 | 3.137 | 1.589 | 0.857 | 0.0639 | -0.092 | 3.271 |
| BMI (normal) | 1.626 | 0.750 | 0.0302 | 0.156 | 3.097 | 2.344 | 0.792 | 0.0031 | 0.791 | 3.897 | 0.159 | 1.059 | 0.8808 | -1.918 | 2.236 | 0.324 | 0.678 | 0.6327 | -1.006 | 1.654 |
| Current health status (good) | 12.814 | 0.922 | <.0001 | 11.006 | 14.622 | 6.537 | 0.973 | <.0001 | 4.628 | 8.446 | 5.985 | 1.301 | <.0001 | 3.432 | 8.538 | 5.052 | 0.833 | <.0001 | 3.417 | 6.687 |
| Neurological problems^c, d^ (yes) | -5.248 | 0.780 | <.0001 | -6.779 | -3.718 | -3.220 | 0.824 | <.0001 | -4.836 | -1.603 | -1.113 | 1.102 | 0.3126 | -3.275 | 1.048 | -1.320 | 0.706 | 0.0617 | -2.704 | 0.065 |
| Presence of comorbidities^d, e^ (yes) | -3.542 | 1.137 | 0.0019 | -5.771 | -1.312 | 0.036 | 1.200 | 0.9760 | -2.318 | 2.390 | 0.291 | 1.605 | 0.8560 | -2.857 | 3.440 | 0.623 | 1.028 | 0.5445 | -1.393 | 2.639 |
| Consultations with a general practitioner^f^ (> 2) | -3.715 | 0.719 | <.0001 | -5.126 | -2.303 | -1.546 | 0.760 | 0.0420 | -3.036 | -0.056 | -0.716 | 1.016 | 0.4809 | -2.709 | 1.277 | -0.438 | 0.651 | 0.5006 | -1.714 | 0.838 |
| Currently smoking (yes) | 1.929 | 1.143 | 0.0916 | -0.312 | 4.171 | -0.060 | 1.207 | 0.9603 | -2.427 | 2.307 | 0.415 | 1.613 | 0.7971 | -2.750 | 3.580 | -1.348 | 1.033 | 0.1924 | -3.375 | 0.680 |
| Current alcohol consumption (yes) | 2.615 | 0.806 | 0.0012 | 1.033 | 4.197 | -0.744 | 0.851 | 0.3821 | -2.414 | 0.926 | 1.241 | 1.138 | 0.2759 | -0.992 | 3.474 | 1.162 | 0.729 | 0.1114 | -0.269 | 2.592 |
| Increased physical activity level (yes) | 2.813 | 0.703 | <.0001 | 1.434 | 4.192 | 1.364 | 0.742 | 0.0663 | -0.092 | 2.820 | 0.708 | 0.993 | 0.4760 | -1.239 | 2.655 | 1.226 | 0.636 | 0.0539 | -0.021 | 2.473 |
| Sleep problems (yes) | -5.219 | 0.821 | <.0001 | -6.829 | -3.608 | -4.333 | 0.867 | <.0001 | -6.034 | -2.632 | -3.268 | 1.160 | 0.0049 | -5.542 | -0.993 | -2.464 | 0.743 | 0.0009 | -3.920 | -1.007 |
| Fatalistic opinion about cancer^g^ (yes) | 0.642 | 0.815 | 0.4312 | -0.957 | 2.241 | 0.426 | 0.861 | 0.6211 | -1.263 | 2.114 | 0.592 | 1.151 | 0.6073 | -1.666 | 2.850 | 0.890 | 0.737 | 0.2277 | -0.556 | 2.336 |
| Brief-COPE: positive thinking^h^ (> 14) | 2.280 | 0.734 | 0.0019 | 0.840 | 3.719 | 6.005 | 0.775 | <.0001 | 4.485 | 7.525 | 6.322 | 1.036 | <.0001 | 4.289 | 8.354 | 1.986 | 0.664 | 0.0028 | 0.685 | 3.288 |
| Brief-COPE: problem solving^h^ (> 11) | 1.022 | 0.724 | 0.1583 | -0.398 | 2.441 | 4.320 | 0.764 | <.0001 | 2.822 | 5.819 | 0.142 | 1.022 | 0.8898 | -1.863 | 2.146 | 0.921 | 0.654 | 0.1594 | -0.362 | 2.205 |
| Brief-COPE: seeking social support^h^ (> 18) | 0.119 | 0.678 | 0.8605 | -1.210 | 1.449 | 2.125 | 0.716 | 0.0030 | 0.721 | 3.529 | 4.337 | 0.957 | <.0001 | 2.460 | 6.215 | 2.200 | 0.613 | 0.0003 | 0.997 | 3.402 |
| Brief-COPE: avoidance^h^ (> 18) | -2.686 | 0.703 | 0.0001 | -4.065 | -1.306 | -5.397 | 0.742 | <.0001 | -6.853 | -3.941 | -3.062 | 0.993 | 0.0021 | -5.009 | -1.114 | -1.143 | 0.636 | 0.0725 | -2.390 | 0.105 |
| MHLCS: internal^h^ (> 22) | 1.471 | 0.687 | 0.0324 | 0.123 | 2.818 | 0.343 | 0.725 | 0.6365 | -1.080 | 1.766 | 0.554 | 0.970 | 0.5683 | -1.349 | 2.456 | 1.185 | 0.621 | 0.0566 | -0.034 | 2.404 |
| MHLCS: powerful others^h^ (> 19) | 0.979 | 0.716 | 0.1718 | -0.426 | 2.384 | 0.779 | 0.756 | 0.3030 | -0.704 | 2.263 | 2.273 | 1.011 | 0.0248 | 0.289 | 4.257 | 0.694 | 0.648 | 0.2844 | -0.577 | 1.964 |
| MHLCS: chance^h^ (> 18) | -0.475 | 0.697 | 0.4953 | -1.842 | 0.891 | -1.218 | 0.736 | 0.0979 | -2.661 | 0.225 | -0.082 | 0.984 | 0.9333 | -2.012 | 1.847 | -0.802 | 0.630 | 0.2031 | -2.038 | 0.434 |
| Health literacy (sufficient) | 0.720 | 0.700 | 0.3037 | -0.653 | 2.094 | 0.810 | 0.739 | 0.2736 | -0.641 | 2.260 | 3.002 | 0.989 | 0.0024 | 1.063 | 4.941 | 1.386 | 0.633 | 0.0288 | 0.144 | 2.628 |
|  |  |  |  |  |  |  |  |  |  |  |  |  |  |  |  |  |  |  |  |  |

^a^ Multiple linear regression model. Models were adjusted for: living status (1: not alone vs. 0: alone); dependents (1: yes vs. 0: no); financial level (1: high vs. 0: low); education level (1: undergraduate to post-graduate degree, 13-17 years vs. 0: high school, ≤ 12 years); professionally active (1: yes vs. 0: no); habitat environment (1: urban vs. 0: rural); age (1: 40-52 years vs. 0: 36-39 years); age (1: 53-75 years vs. 0: 36-39 years); BMI (1: normal vs. 0: overweight or obese); current health status (1: good or very good vs. 0: good enough or lower); neurological problems (1: yes vs. 0: no); presence of comorbidities (1: yes vs. 0: no); consultations with a general practitioner (1: > 2 vs. 0 < 2); currently smoking (1: yes vs. 0: no); current alcohol consumption (1: yes vs. 0: no); increased physical activity level (1: yes vs. 0: no); sleep problems (1: yes vs. 0: no); fatalistic opinion about cancer (1: yes vs. 0: no); Brief-COPE: positive thinking (1: > 14 vs. 0: ≤ 14); Brief-COPE: problem solving (1: > 11 vs. 0: ≤ 11); Brief-COPE: seeking social support (1: > 18 vs. 0: ≤ 18); Brief-COPE: avoidance (1: > 18 vs. 0: ≤ 18); MHLCS: internal (1: > 22 vs. ≤ 22); MHLCS: powerful others (1: > 19 vs. 0: ≤ 19); MHLCS: chance (1: > 18 vs. 0: ≤ 18); health literacy (HLS-EU-Q16) (1: sufficient > 12 vs. 0: limited ≤ 12); current sequelae due to cancer or its treatments (1: yes vs. 0: no); current therapy against cancer (1: yes vs. 0: no); time since diagnosis and questionnaire response (1: 1-3 years vs. 0: 7-10 years); time since diagnosis and questionnaire response (1: 4-6 years vs. 0: 7-10 years); mastectomy (1: yes vs. 0: no); treatment by radiations (1: yes vs. 0: no); drug treatment against cancer (1: yes vs. 0: no);.

^b^ Living with partner or with a family member.

^c^ Neurological problems: Parkinson disease; memory problems requiring consultation; depression, psychological disorders requiring treatment; migraine, or other neurological disease.

^d^ Diagnosis in the last 10 years.

^e^ Comorbidities: cardiovascular disease, neurovascular disease or diabetes.

^f^ In the last 12 months.

^g^ Agree that: “Cancer cannot be avoided”.

^h^ Median value.

* *p* < 0.05; ** *p* < 0.01; *** *p* < 0.0001.
